# Stimulation of adaptive gene amplification by origin firing under replication fork constraint

**DOI:** 10.1093/nar/gkab1257

**Published:** 2022-01-08

**Authors:** Alex J Whale, Michelle King, Ryan M Hull, Felix Krueger, Jonathan Houseley

**Affiliations:** Epigenetics Programme, Babraham Institute, Cambridge, UK; Epigenetics Programme, Babraham Institute, Cambridge, UK; Epigenetics Programme, Babraham Institute, Cambridge, UK; Babraham Bioinformatics, Babraham Institute, Cambridge, UK; Epigenetics Programme, Babraham Institute, Cambridge, UK

## Abstract

Adaptive mutations can cause drug resistance in cancers and pathogens, and increase the tolerance of agricultural pests and diseases to chemical treatment. When and how adaptive mutations form is often hard to discern, but we have shown that adaptive copy number amplification of the copper resistance gene *CUP1* occurs in response to environmental copper due to *CUP1* transcriptional activation. Here we dissect the mechanism by which *CUP1* transcription in budding yeast stimulates copy number variation (CNV). We show that transcriptionally stimulated CNV requires TREX-2 and Mediator, such that cells lacking TREX-2 or Mediator respond normally to copper but cannot acquire increased resistance. Mediator and TREX-2 can cause replication stress by tethering transcribed loci to nuclear pores, a process known as gene gating, and transcription at the *CUP1* locus causes a TREX-2-dependent accumulation of replication forks indicative of replication fork stalling. TREX-2-dependent *CUP1* gene amplification occurs by a Rad52 and Rad51-mediated homologous recombination mechanism that is enhanced by histone H3K56 acetylation and repressed by Pol32 and Pif1. *CUP1* amplification is also critically dependent on late-firing replication origins present in the *CUP1* repeats, and mutations that remove or inactivate these origins strongly suppress the acquisition of copper resistance. We propose that replicative stress imposed by nuclear pore association causes replication bubbles from these origins to collapse soon after activation, leaving a tract of H3K56-acetylated chromatin that promotes secondary recombination events during elongation after replication fork re-start events. The capacity for inefficient replication origins to promote copy number variation renders certain genomic regions more fragile than others, and therefore more likely to undergo adaptive evolution through *de novo* gene amplification.

## INTRODUCTION

Adaptive mutations can enable organisms to tolerate or even thrive in hostile environments. Although all kinds of mutation can be adaptive, CNV - the loss or duplication of segments of genetic material - often underlies adaptation in eukaryotic cells from fungi to mammals (1). Adaptive mutation is frequently reported in chemotherapy resistant cancers and infections (2–7), or treatment resistant animal and plant pests (8,9), so the mechanisms by which adaptive mutations form is of considerable medical, economic and societal interest.

Three major classes of mechanism are implicated in *de novo* CNV (reviewed in (10–12)): firstly, non-allelic homologous recombination either in mitosis or meiosis can occur when a double strand break (DSB) forms within a region homologous to multiple sites in the genome. Strand invasion of the resected DSB into an unmatched homologue may result in duplication, deletion or translocation depending on resolution (reviewed in (13)). Secondly, non-homologous end joining can ligate unmatched DSB ends to create deletions and translocations (reviewed in (14)). Thirdly, replication fork switching between either homologous or microhomologous templates creates discontinuities in the sequence of a daughter chromatid, resulting in CNV or translocations (reviewed in (11) and (15)). All three classes can initiate further complex genome rearrangements by forming unstable species such as dicentric chromosomes or extrachromosomal DNA (reviewed in (12) and (14)), and given that adaptive mutations are normally observed only after extended selection it is often difficult to confirm formation mechanisms.

DNA replication has particular potential to invoke genetic change, and the copious CNV events induced by replication inhibitors such as hydroxyurea or aphidicolin show that non-conservative repair is not an uncommon outcome of replication fork stalling (16–18). Stalled replication forks that cannot be restarted by other means need to be repaired through recombination (19). The simplest model for recombinational repair invokes cleavage of the recombination fork by a structure specific endonuclease (SSE), often Mus81, to create a single ended DSB that can invade the sister chromatid in a process known as Break Induced Replication (BIR) (20,21). Studies using defined nuclease-induced breaks have determined the replication mechanism of BIR: an initial strand invasion mediated by Rad51 creates a D-loop that is extended by DNA polymerase δ (22–25). Replication proceeds in a migrating D-loop rather than a classical replication fork (26,27), with delayed second strand synthesis through copying of the newly synthesized leading strand (27,28). Elongation of the leading strand in the migrating D-loop relies on polymerase δ subunit Pol32, which is dispensable during normal DNA replication, and also the helicase Pif1 (25–27).

Replication forks stalled at an inducible barrier follow a similar but not identical mechanism; replication after restart is performed by DNA polymerase δ and becomes highly error prone (29–31), but the uncoupled semiconservative lagging strand synthesis observed is different to DSB-induced BIR systems and not easily reconciled with the migrating D-loop model (32,33). Indeed, stalled forks can initiate strand invasion via fork reversal without cleavage to form a DSB, which likely explains the difference in elongation mechanism (34,35), although replication fork cleavage by MUS81 and repair by a POLD3 (human Pol32)-dependent BIR mechanism is observed in cancer cells exposed to aphidicolin (36,37). Therefore, repair of stalled replication forks in different organisms and conditions seems to occur through related but non-identical BIR-type mechanisms involving Pol32-dependent synthesis by DNA polymerase δ with uncoupled second strand synthesis and high error rates.

Irrespective of this mechanistic variation, the use of BIR-type mechanisms for replication fork repair seems to be rare and stalled forks are normally resolved by a converging replication fork (38–40). Restricting the use of BIR and related mechanisms makes sense given that BIR-type elongation is prone to mutations (reviewed in (41)), and impaired by encounters with transcribed loci and chromatin marked with Histone 3 Lysine 56 acetylation (H3K56ac) (42,43). Potentially mutagenic BIR-type mechanisms are therefore used only as a last resort, when a converging replication fork does not reach the stalled fork by late G2, and even in this case only minimal chromosomal regions are replicated before encountering a converging fork.

Adaptive mutations (including CNV) emerge through natural selection acting on random mutations. However, all types of mutation have a mechanistic cause that delimits frequency and genomic location, even if the phenotypic outcome of a given mutation is random. Mutation rate in any given genomic window may therefore be constant across time if the environment is constant or if all potentially mutagenic mechanisms acting at that locus are unaffected by environmental change. However, environmental change may disrupt normal DNA processing genome-wide or at specific genomic locations, making use of potentially mutagenic BIR-type mechanisms more frequent. For example, induction of a gene in response to environmental change can impede oncoming replication forks, leading to site specific, environmentally-stimulated mutation ((44,45) and reviewed in (46,47)).

Indeed, we and others have demonstrated that CNV events at the budding yeast *CUP1* locus are stimulated by transcriptional induction of the *CUP1* gene and are tightly localized to the *CUP1* region (48–50). *CUP1* encodes a metallothionein that protects yeast from environmental copper, and the copy number of the *CUP1* gene defines copper resistance such that adaptation to toxic levels of environmental copper occurs primarily through *CUP1* gene amplification (51–53). It is known that RNA polymerase II transcription can impair replication (reviewed in (54)) through head-on collisions between RNA and DNA polymerases (55), generation of torsional stresses (56,57), formation of RNA:DNA hybrids (R-loops) (44,45,58) or formation of secondary DNA structures (59,60), all of which block fork progression and/or cause replication slippage. However, we found that *CUP1* CNV absolutely requires the histone modification Histone 3 Lysine 56 acetylation (H3K56ac) (49), which is not obviously related to any of these outcomes.

Here, we investigate the mechanism by which *CUP1* transcriptional induction causes CNV via H3K56ac, showing critical roles for TREX-2 and Mediator as well as late-firing replication forks adjacent to the *CUP1* genes. We propose a model involving replication origin firing, stalling and collapse that links *CUP1* transcription, errors in BIR and local histone modification to *de novo* CNV.

## MATERIALS AND METHODS

### Yeast strains and media

Yeast strains used are listed in Supplementary Table S1. Deletion strains were produced by standard deletion protocols using oligonucleotides listed in Supplementary Table S2 and validated by PCR. Construction of 3x*CUP1* new ARS and control strains: fragments of pRS316 containing *URA3* region ± ARS were amplified using oligonucleotides listed in Supplementary Table S2 and integrated in YRH23. Construction of *3xCUP1* no ARS strain: pJH285 containing one repeat *GFP-CUP1* was formed by ligating the XmaI BglII-(blunt) fragment of pFA6a-GFP-KanMX6 into pJH254 (49) digested with XmaI EcoRV. The three repeat plasmid pJH287 was formed by ligating three fragments—pJH285 ClaI SalI, pJH285 XhoI BglII and pJH285 BamHI EcoRI—simultaneously into EcoRI ClaI digested pJH264 (49). This construct was integrated into genome of YRH15 as described in (49).

All cells were cultured in shaking incubators at 30°C, 200 rpm. Overnight cultures and cells used in standard experiments were grown in yeast nitrogen base (YNB) media (which contains 250 nM CuSO_4_) that was supplemented with CSM amino acids and 2% glucose (or 2% raffinose with 0.02% galactose when stated). YNB media and supplements were purchased from Formedium. Pre-cultures for all copper experiments were grown to saturation (∼2 days) in 4 ml cultures of YNB media and then diluted 1:2000 for subsequent treatments. For nicotinamide treatment, cells were cultured for one week in 4 ml YNB media with a final concentration of 5 mM Nicotinamide (Sigma I17451). For copper treatment, cells were grown in 4 ml YNB media ± 0.3 mM CuSO_4_ for 1 week. For northern blot analysis, cells were grown in 4 ml YNB media with 2% glucose for 6 h, diluted and grown overnight in 25 ml same media to 0.6–0.8 × 10^7^ cells/ml. Un-induced cells were harvested, cells were diluted to 0.15 × 10^7^ cells/ml in 25 ml with 0.3 mM CuSO_4_ and grown for 6 h before harvesting 2 × 10^7^ cells by centrifugation and freezing on N_2_. For TrAEL-seq experiments, cells were pre-cultured by inoculation in 4 ml yeast peptone broth containing 2% raffinose (YP Raf) for ∼6 h at 30°C with shaking at 200 rpm. These cells were then diluted in 100 ml YP Raf (wild-type and *rad52*Δ cells ∼1:500, *sac3*Δ cells ∼1:50) and growth continued at 30°C 200 rpm for ∼16 h until OD_600_ reached ∼0.2. These 100 ml cultures were then split, with 50 ml transferred into 50 ml YP Raf media and the other 50 ml being transferred into 50 ml YP Raf media containing 0.02% galactose for 6 h at 30°C 200 rpm. Cells were centrifuged 1 min at 4600 rpm, resuspended in 70% ethanol at 1 × 10^7^ cells/ml and stored at −70°C. YP media, raffinose and galactose were purchased from Formedium.

### Adaptation assay

From saturated cultures grown ±0.3 mM CuSO_4_, a 1:80 dilution in 200 μl of YNB media was placed in every well in a flat-bottomed 96-well cell culture plate with CuSO_4_ at the required concentration. Plates were sealed using a gas-permeable membrane and incubated at 30°C with shaking for 3 days. Cells were resuspended and OD_660_ was measured by a BD FLUOstar Omega plate reader. Area-Under-Curve for plots of OD_660_ against [CuSO_4_] were calculated for each sample and compared by one way ANOVA with Sidak's multiple comparison correction in GraphPad Prism (v8.2.1).

### DNA extraction and Southern blotting

From a saturated culture, 2 ml of cells were washed in 50 mM EDTA and then spheroplasted using 250 μl of 0.34 U/ml lyticase (Sigma L4025) in 1.2 M sorbitol, 50 mM EDTA and 10 mM DTT at 37°C for 45 min. These cells were centrifuged at 1000 rcf, gently resuspended in 400 μl of 100 μg/ml RNase A (Sigma R4875), 50 mM EDTA and 0.3% SDS, and then incubated for 30 min at 37°C. After this, 4 ul of 20 mg/ml proteinase K (Roche 3115801) was added, mixed by inverting the samples and heated at 65°C for 30 min. The samples were then left to cool to room temperature before adding 160 μl of 5 M KOAc, then mixed by inversion and chilled on ice for 1 h. These samples were centrifuged at 20 000 rcf for 10 min before the supernatant was poured into a new tube containing 500 μl of phenol:chloroform (pH8) and placed on a rotating wheel for 30 min. After centrifugation at 10 000 rcf for 10 min, the upper phase was extracted using wide bore pipette tips and precipitated in 400 μl isopropanol. Pellets were then washed in 70% ethanol, left to air-dry and then digested overnight at 37°C in 50 μl TE with 20 U EcoRI-HF (NEB). Samples were extracted with 50 μl phenol:chloroform, then ethanol precipitated in a 1.5 ml tube containing 112.5 μl 100% ethanol and 4.5 μl 3 M NaOAc before centrifugation at 20 000 rcf for 15 min. After washing in 70% ethanol, the pellets were dissolved for 1 h in 20 μl TE. Loading dye was added and samples were separated on 25 cm 0.8% or 1% TBE gels at 120 V for 16.5 h. Gels were denatured in 0.25 N HCl for 15 min, neutralized in 0.5 N NaOH for 45 min and washed twice in 1.5 M NaCl, 0.5 M Tris (pH 7.5) for 20 min each. Samples were transferred to HyBond N + membrane in 6× SSC through capillary action overnight and fixed by UV crosslinking using a Stratagene UV Stratalinker. Membranes were probed using random primed probes (listed in Supplementary Table S2) in 10 ml UltraHyb (AM8669 ThermoFisher Scientific) at 42°C then washed with 0.1× SSC 0.1% SDS at 42°C. Quantification of Southern blot bands was performed using ImageQuant (Version 7.0, GE), and CNV calculated as (intensity of all CNV bands/intensity of CNV and parental bands) × 100. Statistical analysis of CNV levels was performed by one-way ANOVA with Sidak's multiple comparison correction in GraphPad Prism (v8.2.1).

### RNA extraction and northern blotting

Frozen cell pellets were lysed by 5 min vortexing at 4°C with ∼50 μl glass beads and 40 μl GTC-phenol (2.1 M guanidine thiocyanate, 26.5 mM Na citrate pH 7, 5.3 mM EDTA, 76 mM β-mercaptoethanol, 1.06% *N*-lauryl sarcosine, 50% phenol pH 7). 600 μl GTC-phenol was added, mixed, and samples were heated at 65°C for 10 min then placed on ice for 10 min. 160 μl 100 mM NaOAc pH 5.2 and 300 μl chloroform:isoamyl alcohol (24:1) were added, samples were vortexed and centrifuged at top speed for 5 min. The upper phase was re-extracted first with 500 μl phenol:chloroform pH 7 (1:1) and then with 500 μl chloroform:isoamyl alcohol (24:1) before precipitation with 1 ml ethanol. Pellet was washed with 70% ethanol, re-suspended in 6 μl water and quantified using Quant-IT RiboGreen (ThermoFisher, R11490). 1 μg RNA was resolved per lane on 1.2% glyoxal agarose gels, blotted and probed with a random primed probe against *CUP1* ORF (Supplementary Table S2) as described (61).

### Candidate genetic screen

A total of 206 strains from the Yeast Deletion Collection (Invitrogen 95401.H2) and other sources were streaked out on YPD agar plates and then re-streaked for single colonies on YPD plates containing 300 μg/ml G418. Pre-cultures were grown to saturation in 4 ml YNB media at 30°C, diluted 1:2000 in 4 ml YNB media with and without 5 mM Nicotinamide and then incubated for 1 week at 30°C with shaking. DNA extraction and Southern blotting was performed as described above. CNV rates of mutants were obtained by comparing the percentage of CNV alleles in nicotinamide-treated mutants to nicotinamide-treated wildtype cells, and calculating the fold change in CNV.

For network analysis, factors from the CNV screen and their first neighbours were imported into Cytoscape (v3.7.2) using stringApp (v1.6.0) (62) to retrieve *Saccharomyces cerevisiae* protein interaction data and to construct the network. The Edge-weighted Spring Embedded layout was applied using ‘stringdb score’ to determine edge length, and node size and colour were mapped to fold-change in CNV from the CNV screen with labels applied to the strongest CNV enhancers and suppressors (>2 or <0.5 fold-change in CNV, respectively). Cluster Analysis was performed on this network using the ClusterONE app (v1.0) (63) which was used to identify clusters of proteins, and clusters with a similar impact on fold-change in CNV have their functional categories displayed.

### TrAEL-seq library preparation and sequencing

1–3 × 10^7^ cells fixed in ethanol were pelleted by centrifugation for 30 s at 20 000 g, rinsed in 1 ml PFGE wash buffer (10 mM Tris HCl pH 7.5, 50 mM EDTA), and resuspended in 60 μl PFGE wash buffer containing 1 μl lyticase (17 U/μl 10 mM KPO_4_ pH 7, 50% glycerol Merck L2524 > 2000 U/mg) then incubated for 10 min at 50°C. 40 μl of molten CleanCut agarose (Bio-Rad 1703594) cooled to 50°C was added, samples were vortexed vigorously for 5 s and pipetted into a plug mould (Bio-Rad 1703713), then left to solidify for 30 min at 40°C. Plugs were transferred into a 2 ml Eppendorf that contained 500 μl PFGE wash buffer containing 10 μl 17 U/ml lyticase and left to incubate at 37°C for 1 h. This solution was removed and replaced with 500 μl PK buffer (100 mM EDTA pH 8, 1 mg/ml Proteinase K, 1% sodium *N*-lauroyl sarcosine, 0.2% sodium deoxycholate) at 50°C overnight. The plugs were then rinsed in 1 ml TE and washed with 1 ml TE for 1 h with rocking. Plugs were then washed twice with 1 ml TE containing 10 mM PMSF (Merck 93482) for 1 h with rocking. Finally, plugs were digested in 200 μl TE containing 1 μl 1000 U/ml RNase T1 (Thermo EN0541) at 37°C for 1 h before being stored at 4°C in 1 ml TE.

A $\frac{1}{2}$ plug was used for each sample (referred to here on in as plugs). Plugs were equilibrated once in 100 μl 1× TdT buffer (NEB) for 30 min at room temperature, then incubated for 2 h at 37°C in 100 μl 1× TdT buffer containing 4 μl 10 mM ATP and 1 μl Terminal Transferase (NEB M0315L). Plugs were rinsed with 1 ml tris buffer (10 mM Tris–HCl pH 8.0), equilibrated in 100 μl 1× T4 RNA ligase buffer (NEB) containing 40 μl 50% PEG 8000 for 1 h at room temperature then incubated overnight at 25°C in 100 μl 1× T4 RNA ligase buffer (NEB) containing 40 μl 50% PEG 8000, 1 μl 10 pM/μl TrAEL-seq adaptor 1 (64) and 1 μl T4 RNA ligase 2 truncated KQ (NEB M0373L). Plugs were then rinsed with 1 ml tris buffer, transferred to 15 ml tubes and washed three times in 10 ml tris buffer with rocking at room temperature for 1–2 h each, then washed again overnight under the same conditions. Plugs were equilibrated for 15 min with 1 ml agarase buffer (10 mM Bis–Tris–HCl, 1 mM EDTA pH 6.5), then the supernatant removed and 50 μl agarase buffer added. Plugs were melted for 20 min at 65°C, transferred for 5 min to a heating block pre-heated to 42°C, 1 μl β-agarase (NEB M0392S) was added and mixed by flicking without allowing sample to cool, and incubation continued at 42°C for 1 h. DNA was ethanol precipitated with 25 μl 10 M NH_4_OAc, 1 μl GlycoBlue, 330 μl of ethanol and resuspended in 10 μl 0.1× TE. 40 μl reaction mix containing 5 μl Isothermal amplification buffer (NEB), 3 μl 100 mM MgSO_4_, 2 μl 10 mM dNTPs and 1 μl Bst 2 WarmStart DNA polymerase (NEB M0538S) was added and sample incubated 30 min at 65°C before precipitation with 12.5 μl 10 M NH_4_OAc, 1 μl GlycoBlue, 160 μl ethanol and re-dissolving pellet in 130 μl 1× TE. The DNA was transferred to an AFA microTUBE (Covaris 520045) and fragmented in a Covaris E220 using duty factor 10, PIP 175, Cycles 200, Temp 11°C, then transferred to a 1.5 ml tube containing 8 μl pre-washed Dynabeads MyOne streptavidin C1 beads (Thermo, 65001) re-suspended in 300 μl 2× TN (10 mM Tris pH 8, 2 M NaCl) along with 170 μl water (total volume 600 μl) and incubated 30 min at room temperature on a rotating wheel. Beads were washed once with 500 μl 5 mM Tris pH 8, 0.5 mM EDTA, 1 M NaCl, 5 min on wheel and once with 500 μl 0.1× TE, 5 min on wheel before re-suspension in 25 μl 0.1× TE. Second end processing and library amplification were performed with components of the NEBNext Ultra II DNA kit (NEB E7645S) and a NEBNext Multiplex Oligos set (e.g. NEB E7335S). 3.5 μl NEBNext Ultra II End Prep buffer, 1 μl 1 ng/μl sonicated salmon sperm DNA (this is used as a carrier) and 1.5 μl NEBNext Ultra II End Prep enzyme were added and reaction incubated 30 min at room temperature and 30 min at 65°C. After cooling, 1.25 μl 10 pM/μl TrAEL-seq adaptor 2 (64), 0.5 μl NEBNext ligation enhancer and 15 μl NEBNext Ultra II ligation mix were added and incubated 30 min at room temperature. The reaction mix was removed and discarded and beads were rinsed with 500 μl wash buffer (5 mM Tris pH 8, 0.5 mM EDTA, 1 M NaCl) then washed twice with 1 ml wash buffer for 10 min on wheel at room temperature and once for 10 min with 1 ml 0.1× TE. Libraries were eluted from beads with 11 μl 1× TE and 1.5 μl USER enzyme (NEB) for 15 min at 37°C, then again with 10.5 μl 1× TE and 1.5 μl USER enzyme (NEB) for 15 min at 37°C, and the two eluates combined. An initial test amplification was used to determine the optimal cycle number for each library. For this, 1.25 μl library was amplified in 10 μl total volume with 0.4 μl each of the NEBNext Universal and any NEBNext Index primers with 5 μl NEBNext Ultra II Q5 PCR master mix. Cycling program: 98°C 30 s then 18 cycles of (98°C 10 s, 65°C 75 s), 65°C 5 min. Test PCR was cleaned with 8 μl AMPure XP beads (Beckman A63881) and eluted with 2.5 μl 0.1× TE, of which 1 μl was examined on a Bioanalyser high sensitivity DNA chip (Agilent 5067-4626). Ideal cycle number should bring final library to final concentration of 1–3 nM, noting that the final library will be 2–3 cycles more concentrated than the test anyway. 21 μl of library was then amplified with 2 μl each of NEBNext Universal and chosen Index primer and 25 μl NEBNext Ultra II Q5 PCR master mix using same conditions as above for calculated cycle number. Amplified library was cleaned with 40 μl AMPure XP beads (Beckman A63881) and eluted with 26 μl 0.1× TE, then 25 μl of this was again purified with 20 μl AMPure XP beads and eluted with 11 μl 0.1× TE. Final libraries were quality controlled and quantified by Bioanalyser (Agilent 5067-4626) and KAPA qPCR (Roche KK4835). Libraries were sequenced on an Illumina NextSeq 500 as High Output 75 bp Single End by the Babraham Institute Next Generation Sequencing facility.

### TrAEL-seq data processing

Unique Molecular Identifier (UMI) deduplication and mapping: Scripts used for UMI-handling as well as more detailed information on the processing are available here: https://github.com/FelixKrueger/TrAEL-seq). Briefly, TrAEL-seq reads carry an 8 bp in-line barcode (UMI) at the 5′-end, followed by a variable number of 1–3 thymines (T). Read structure is therefore NNNNNNNN(T)nSEQUENCESPECIFIC, where NNNNNNNN is the UMI, and (T)n is the poly(T). The script TrAELseq_preprocessing.py removes the first 8 bp (UMI) of a read and adds the UMI sequence to the end of the readID. After this, up to 3 T (inclusive) at the start of the sequence are removed. Following this UMI and Poly-T pre-processing, reads underwent adapter- and quality trimming using Trim Galore (v0.6.5; default parameters; https://github.com/FelixKrueger/TrimGalore). UMI-pre-processed and adapter-/quality trimmed files were then aligned to the respective genome using Bowtie2 (v2.4.1; option: –local; http://bowtie-bio.sourceforge.net/bowtie2/index.shtml) using local alignments. Finally, alignment results files were deduplicated using UmiBam (v0.2.0; https://github.com/FelixKrueger/Umi-Grinder). This procedure deduplicates alignments based on the mapping position, read orientation as well as the UMI sequence.

To assist with the interpretation of aligned multi-copy sequences, the *P_GAL1_-HA cup1* samples were treated in a more specialized way before entering the TrAEL-seq processing procedure outlined above: Prior to TrAEL-seq pre-processing, sequences were deduplicated based on the first 23 bp on their 5′-end (using the script TrAELseq_sequence_based_deduplication.py). This region contains both the UMI sequence as well as the first 15 bp of genomic sequence, and should thus help identify (and remove) PCR amplified multi-copy sequences that would under normal conditions survive the UMI-aware deduplication procedure by aligning to several different genomic regions at random. Following deduplication-by-sequence and TrAEL-seq pre-processing, these sequences were aligned to a modified version of the yeast genome containing the additional P_gal_-HA control sequences. To avoid multi-mapping artefacts arising from the integration of these sequences, the following two stretches of genomic sequence were masked by Ns: a) *CUP1* (chromosome VIII:212266–216251), and b) *P_GAL1_* (chromosome II:278352–279023). Reads were then trimmed and mapped as above.

De-duplicated mapped reads were imported into SeqMonk v1.47 (https://www.bioinformatics.babraham.ac.uk/projects/seqmonk/) and immediately truncated to 1 nucleotide at the 5′ end, representing the last nucleotide 5′ of the strand break. Reads were then summed in running windows as described in figure legends. Windows overlapping with non-single copy regions of the genome were filtered (rDNA, 2μ, mtDNA, sub-telomeric regions, Ty elements and LTRs), and total read counts across all included windows were normalized to reads per million mapped. A further enrichment normalization (20–90%) was applied to match the read count distributions of the *P_GAL1_-HA cup1* libraries. Read counts were exported for the consensus *P_GAL1_-HA cup1* region and plotted in GraphPad Prism 8. Comparison of datasets was performed using edgeR implemented in SeqMonk (65). For read polarity plots, forward and reverse read counts were quantitated in running windows as specified in the relevant figure legends before export for plotting using R v4.0.0 in RStudio. Read polarity values were calculated and plotted as either dots (individual samples) or as a continuous line (multiple sample display) for each quantification window using the formula read polarity = (R – F)/(R + F), where F and R relate to the total forward and reverse read counts respectively. The R code to generate these plots can also be found here: https://github.com/FelixKrueger/TrAEL-seq.

### Image processing

Gel images were processed in ImageQuant TL (v7.0) which involved cropping, rotating and altering contrast of whole images to improve visualization of bands.

## RESULTS

### A genetic screen identifies enhancers and suppressors of *CUP1* CNV

**Figure 1.**
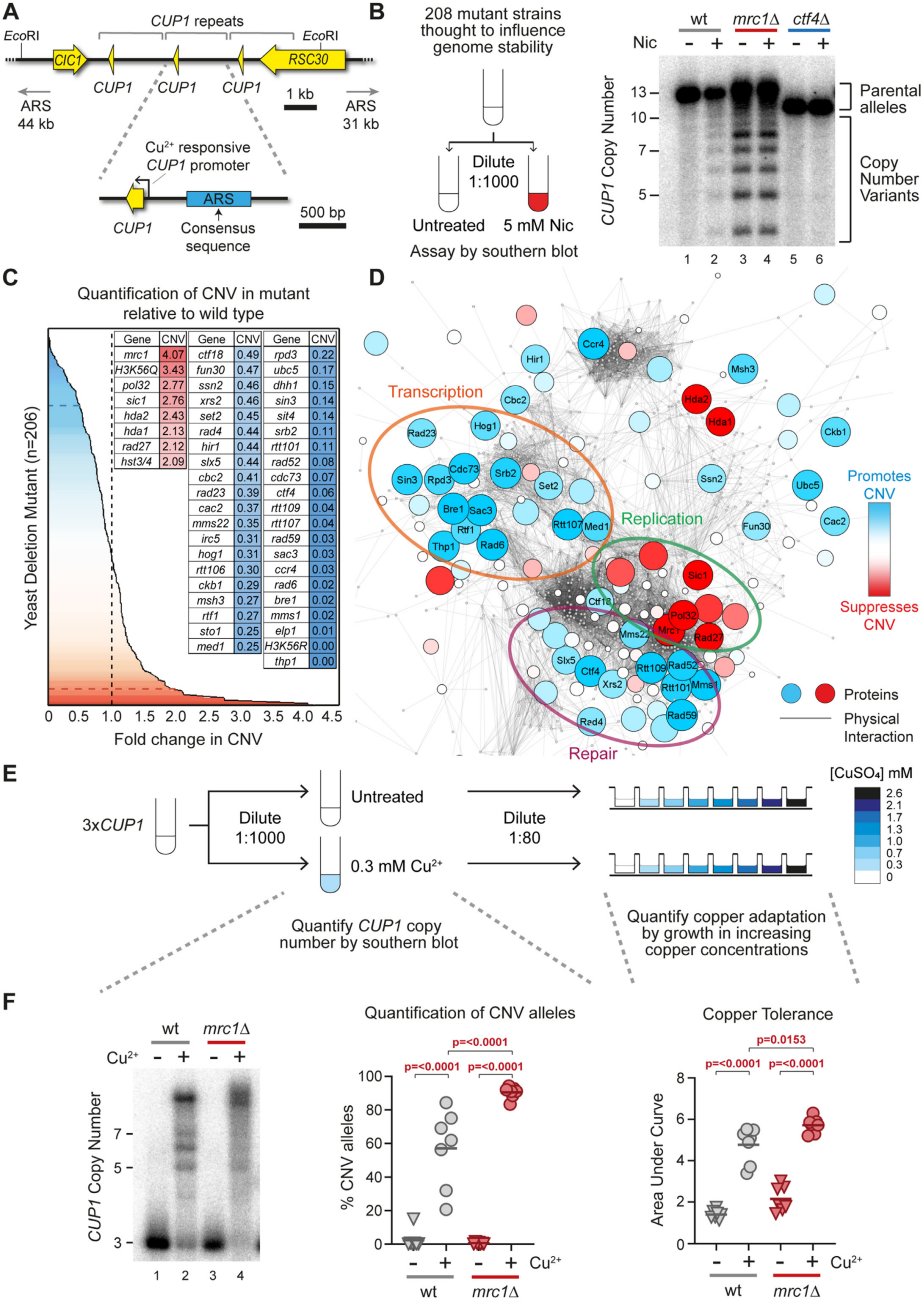
A screen for genes regulating transcriptionally-stimulated CNV. (**A**) Schematic of the *CUP1* array and the surrounding region of Chromosome VIII. ORFs are shown in yellow, and grey brackets indicate the repeated region. Each repeat contains a replication origin (ARS); the ARS consensus sequence was defined in (124) and the blue rectangle denotes a sequence with ARS activity experimentally validated in (104) though the actual limits of the ARS element have not been determined. Schematic shows cells with three copies of the *CUP1* gene although the BY4741 background used as in the initial screen contains 13 *CUP1* copies. The nearest adjacent replication origins (ARS elements) in both directions are indicated. (**B**) Schematic of the candidate genetic screen for regulators of CNV and a representative Southern blot used for quantification of CNV. Wild type (wt) and indicated mutant cells from yeast haploid deletion collection (Invitrogen 95401.H2) and other sources (Supplementary Table S3) were grown to saturation then diluted 1:1000 and re-grown to saturation (10 generations) ±5 mM nicotinamide (Nic) and subject to southern analysis of *CUP1* copy number. Quantification of % CNV alleles was calculated as the percentage of alleles deviating from the parental copy number and fold-change was calculated relative to % CNV alleles in nicotinamide-treated wild-type cells. (**C**) Summary of genetic screen plotting fold-change in nicotinamide-induced CNV of 206 deletion strains relative to wild-type. Grey dashed line indicates wild-type fold-change in CNV. The blue dashed line represents cut-off for CNV-suppressing mutations with a <0.5 fold change in CNV and red dashed lines indicates the cut-off for CNV enhancing mutations with a >2-fold change in CNV. Genes called as enhancers (blue) or suppressors (red) are shown in inset table, full results are given in Supplementary Table S3. (**D**) Protein–Protein Physical Interaction Network of factors from the CNV screen and their first neighbours (visualized in Cytoscape Version 3.7.2). Nodes represent proteins and edges represent high-confidence physical interactions between proteins imported from stringApp (v1.6.0). The size and colour of nodes indicates deviation from wild-type fold-change in CNV, with red nodes representing increasing rates of CNV in mutant and blue nodes representing decreasing rates of CNV in mutant. First neighbours of screen factors are shown as small grey nodes. Clusters of interacting proteins (identified in the physical interaction network using ClusterONE v1) which showed similar fold-change in CNV are circled. (**E**) Methodology for assessing CNV and copper adaptation in cells containing three copies of the *CUP1* gene (*3xCUP1*). Saturated cultures of 3x*CUP1* wild-type and mutant cells were diluted 1:1000 in media ±0.3 mM CuSO_4_ and incubated for 1 week at 30°C. Half the culture was used for Southern blot analysis, while the other half was diluted 1:80 and grown in a 96-well plate containing a range of concentrations of CuSO_4_ to assay for copper tolerance. (**F**) Example southern blot analysis of *CUP1* locus and copper tolerance assay in wild type (wt) and *mrc1*Δ mutant strain with three copies of the *CUP1* gene, as outlined in (E); Southern blot quantification shows the percentage of alleles deviating from the parental copy number, for adaptation OD_600_ was plotted against [CuSO_4_] and copper tolerance quantified as area-under-curve for each culture; *n* = 7, *P*-values calculated by one-way ANOVA.

The *CUP1* locus on chromosome VIII is composed of 1 or more tandem copies of a 2 kb sequence containing the *CUP1* gene with a copper responsive *CUP1* promoter and a poorly defined replication origin (an autonomously replicating sequence or ARS) (Figure 1A). Transcriptional activation of the locus promotes *de novo* CNV events including gene amplifications that increase copper resistance, such that exposure of budding yeast to copper-rich environments leads to the formation of new *CUP1* alleles that improve resistance (49).

To identify factors important for *CUP1* CNV, we performed a candidate genetic screen using 208 mutants drawn largely from the Yeast Deletion Collection (66), but with selected double deletion mutants, hypomorphs and histone point mutants (see Supplementary Table S3 for data on all strains tested). Mutants were selected to survey a wide range of mechanisms involved in RNA processing, DNA replication, DNA repair and genome stability. The S288C genetic background of these mutants includes a 13 copy *CUP1* array that rarely amplifies but does undergo transcriptionally stimulated contraction through an H3K56ac-dependent mechanism (49). Nicotinamide, an inhibitor of H3K56 deacetylases, accentuates CNV stimulation by transcription, such that even basal *CUP1* expression in the absence of copper becomes sufficient to cause measurable contractions (49). Fold-change in CNV relative to wild type can then be quantified by Southern blot after 10 generations ± nicotinamide to reveal enhancers or suppressors of CNV such as *MRC1* or *CTF4* (Figure 1B).

Seven deletion mutants exhibited a >2-fold increase in CNV, including four DNA replication mutants (*mrc1*Δ, *pol32*Δ, *sic1*Δ and *rad27*Δ) and three histone deacetylase mutants (*hda1*Δ, *hda2*Δ and *hst3*Δ *hst4*Δ). Forty-one mutants suppressed CNV >2-fold, representing a wider range of biological processes including regulation of transcription (*thp1*Δ, *med1*Δ, *cdc73*Δ etc.), DNA repair (*rtt107*Δ, *rad59*Δ, *rad52*Δ etc.), nucleosome assembly (*rtt106*Δ, *cac2*Δ, *hir1*Δ etc.) and various histone modifiers (*bre1*Δ, *rtt109*Δ, *rpd3*Δ etc.) (Figure 1C). ClusterONE, which identifies clusters of interacting proteins in a physical interaction network, identified clusters of proteins that have similar effects on CNV. Clusters of proteins that promote CNV are involved in chromatin/transcription (orange circle) and DNA repair (purple circle), while a cluster of proteins that facilitate DNA replication & cell cycle progression all tend to suppress CNV (green circle) (Figure 1D). These clusters are coherent with the genetic dependencies we have previously observed (49).

To validate the importance of these genes in adaptive *CUP1* amplification, selected genes were deleted in a tester strain that carries only three copies of *CUP1* (3x*CUP1*) (Figure 1E and Supplementary Figure S1). During growth in sub-lethal copper, 3x*CUP1* cells that undergo *CUP1* amplification gain a selective advantage and become dominant in the population. *CUP1* amplification allows growth in higher concentrations of copper, so cultures grown in sub-lethal copper acquire increased resistance as cells with amplified alleles proliferate. These phenotypes can be quantified by Southern blot and copper resistance assays respectively (Figure 1F). For example, deletion of *MRC1* in the 3x*CUP1* background increases the proportion of cells that acquire adaptive *CUP1* amplifications over 10 generations growth in sub-lethal copper, which increases adaptation of the population to higher concentrations of copper.

Therefore, our candidate genetic screen for enhancers and suppressors of *CUP1* CNV provides a genetic profile consistent with a replication-linked recombination mechanism driven by transcription.

### TREX-2 and Mediator are required for transcriptionally stimulated CNV

**Figure 2.**
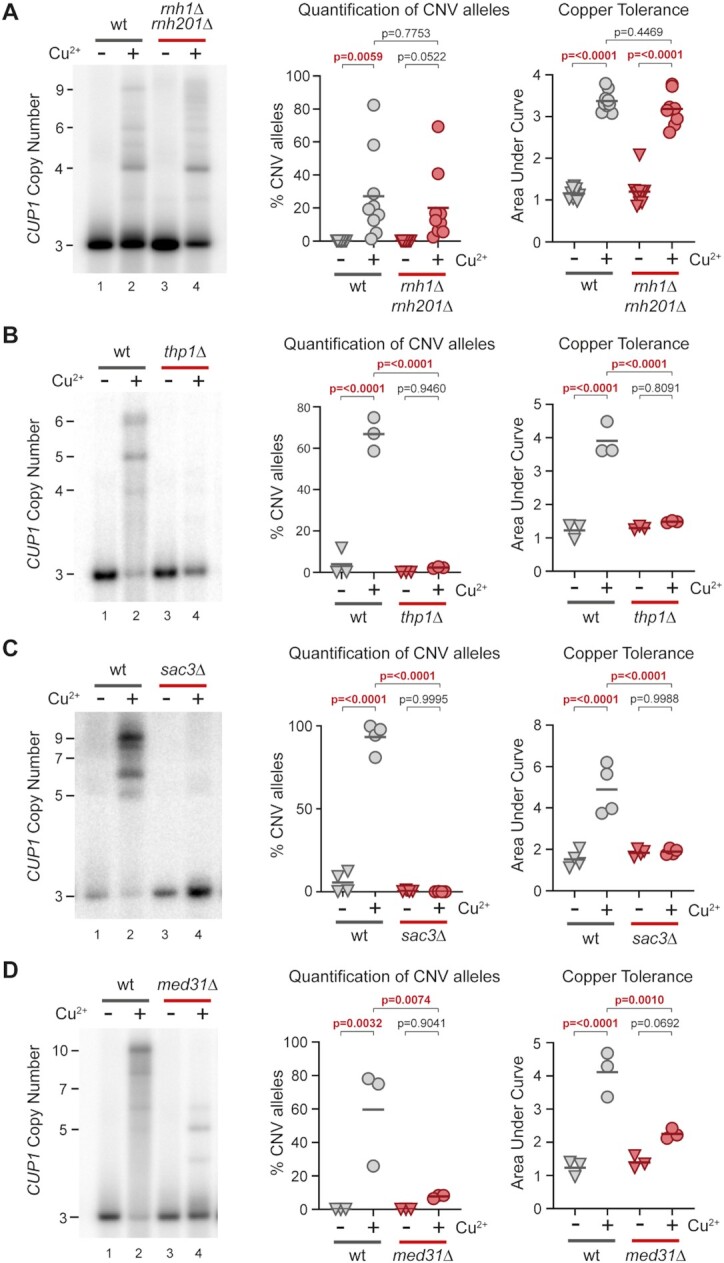
TREX-2 and Mediator are required for transcription stimulated *CUP1* CNV. (**A**) Southern blot analysis of *CUP1* copy number in wild-type (wt) and *rnh1*Δ *rnh201*Δ cells with three copies of the *CUP1* gene, grown to saturation in YNB media ±0.3 mM CuSO_4_; *n* = 9. Quantification shows the percentage of alleles deviating from the parental copy number. Copper adaptation was assessed by diluting these same cells in 96-well plates with varying concentrations of CuSO_4_ and incubating for 3 days (see Figure 1E). Final OD_600_ was plotted against [CuSO_4_] and copper tolerance quantified as area-under-curve for each culture; all *P*-values were calculated by one-way ANOVA. (**B**) Southern analysis of *CUP1* copy number in *3xCUP1* wild-type (wt) and *thp1*Δ cells after 10 generations ±0.3 mM CuSO_4_ (Quantification of CNV alleles and Copper adaptation as in A); *n* = 3. (**C**) Southern analysis of *CUP1* copy number in *3xCUP1* wild-type (wt) and *sac3*Δ cells after 10 generations ±0.3 mM CuSO_4_ (quantification of CNV alleles and copper adaptation as in A); *n* = 4. (**D**) Southern analysis of *CUP1* copy number in *3xCUP1* wild-type (wt) and *med31*Δ cells after 10 generations ±0.3 mM CuSO_4_ (Quantification of CNV alleles and copper adaptation as in A); *n* = 3.

Many recent studies have shown that RNA:DNA hybrids called R-loops impair replication fork progression, providing a well-validated mechanism for transcription-associated recombination ((67), reviewed in (68)). In consequence, we expected mutants affecting R-loop formation or processing to show strong phenotypes in the genetic screen, but *hpr1*Δ, *tho2*Δ and *thp2*Δ cells (THO Complex mutants) which accumulate R-loops had little effect (69–71) (Supplementary Table S3). Furthermore, *rnh1*Δ *rnh201*Δ cells known to accumulate high levels of R-loops underwent copper adaptation and *CUP1* CNV at normal rates in the 3x*CUP1* background (Figure 2A) (72–74). We therefore find no evidence that R-loops are involved in transcriptionally stimulated *CUP1* CNV.

In contrast, two of the strongest suppressor mutants found in the screen were deletions of *SAC3* and *THP1*, which encode components of Transcription and RNA Export complex 2 (TREX-2) (75,76). Remarkably, 3x*CUP1 thp1*Δ and *3xCUP1 sac3*Δ mutants underwent no detectable *CUP1* CNV and were completely unable to adapt to copper despite showing normal resistance to sub-lethal concentrations of copper (Figure 2B, C and Supplementary Figure S2A). Although TREX-2 mutations alter expression of some genes, we did not detect any difference in the induction of *CUP1* in response to copper (Supplementary Figure S2B) (77,78). This implicates TREX-2 as a link between transcription and recombination events at *CUP1*.

TREX-2 is physically associated with the Mediator complex (77) and mediator mutant *med1*Δ also suppressed *CUP1* CNV in the genetic screen. Med1 is a component of the middle module of Mediator but has not been placed within the structure (reviewed in (79)), whereas the TREX-2/Mediator interface has been mapped to Med31, which projects out from the middle module (77,80,81). Just as we observed in TREX-2 mutants, 3x*CUP1 med31*Δ cells did not undergo detectable CNV or adaptation during growth in sub-lethal copper, despite normal *CUP1* mRNA induction (Figure 2D and Supplementary Figure S2B). We further analysed cells lacking Srb2, a component of the head module located at the interface between Mediator and RNA polymerase II (82,83), and found that 3x*CUP1 srb2*Δ cells were similarly impaired both in *CUP1* amplification and acquisition of increased copper resistance (Supplementary Figure S2C). These results reveal a critical role for both TREX-2 and Mediator in the mechanism of *CUP1* amplification.

Mediator connects transcription factors to the core RNA polymerase II machinery at active promoters (reviewed in (84)), while TREX-2 associates with nuclear pores to promote mRNA processing and export (85). However, the gene expression and RNA export functions of Mediator and TREX-2 are separable (77), so the requirement for both complexes in *CUP1* amplification implicates their shared physical interaction. This interaction connects actively transcribed genes to the nuclear pore in a process termed gene gating (77,86–88). Gene gating can impair DNA replication by constraining DNA topology, causing replication forks rendered already fragile by HU treatment to collapse in a Rad53 mutant lacking checkpoint activity (56). This process, albeit only previously characterized under considerable replicative stress, provides a mechanism by which *CUP1* transcriptional induction acting through TREX-2 and Mediator could impede replication forks without a requirement for R-loop formation.

### Evidence for replication fork stalling caused by *cup1* transcription

**Figure 3.**
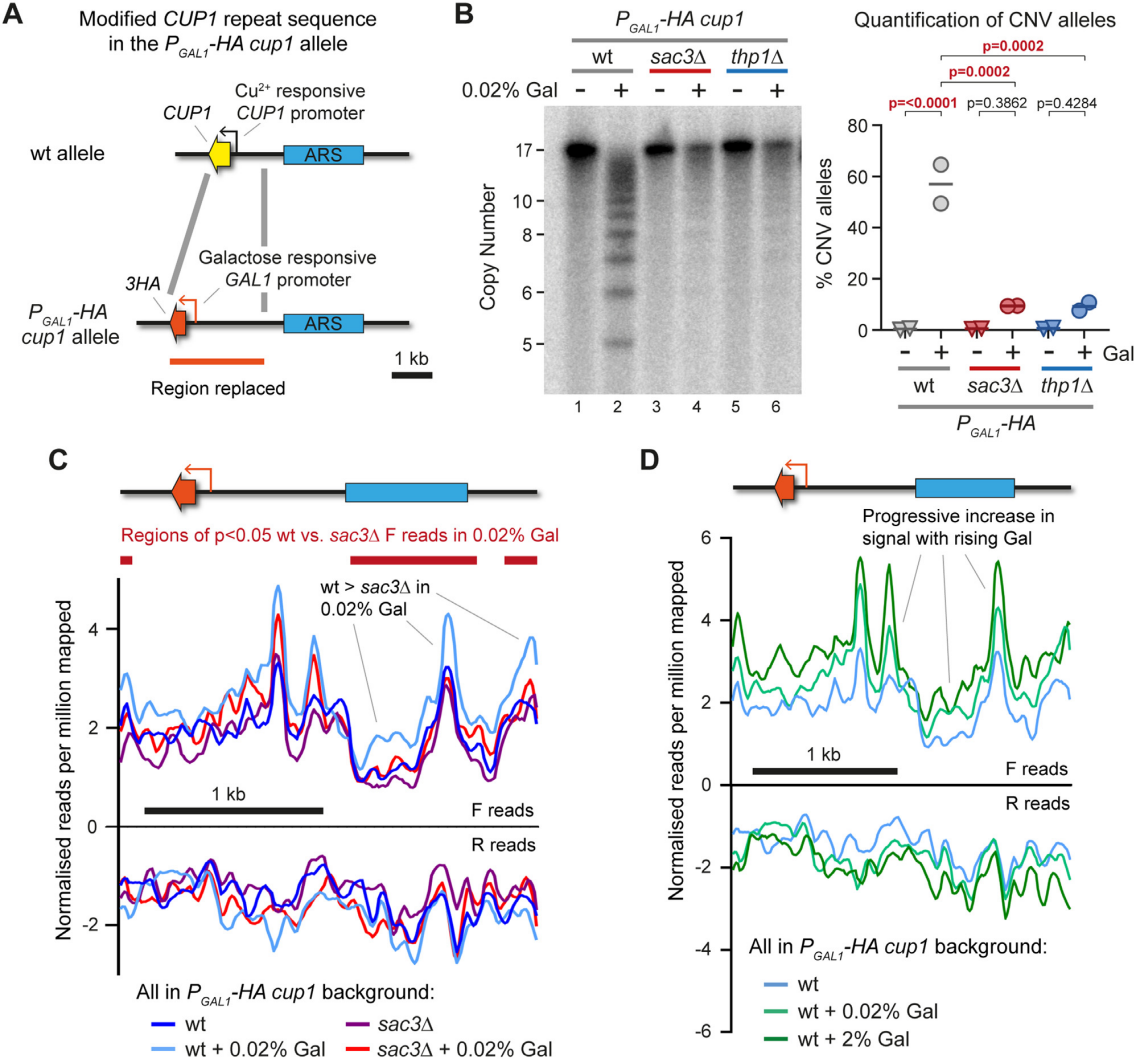
Replication fork stalling and cleavage at *CUP1* locus. (**A**) Schematic of modified *CUP1* repeat in *P_GAL1_-HA cup1* cells, where every *CUP1* ORF is replaced by a 3HA coding sequence (which is phenotypically neutral) and every *CUP1* promoter is replaced by the galactose-inducible *GAL1* promoter. (**B**) Southern blot analysis of *P_GAL1_-HA cup1* cells comparing *sac3*Δ and *thp1*Δ cells to wild-type cells grown for 10 generations in 2% raffinose ±0.02% galactose. Quantification shows the percentage of alleles deviating from the parental copy number of 17 copies; p-values were calculated by one-way ANOVA, *n* = 2. (**C**) Plots of TrAEL-seq read density on forward and reverse strands across a *P_GAL1_-HA cup1* repeat. Forward TrAEL-seq reads in replicating cells arise primarily from replication forks moving right-to-left, reverse reads from forks moving left-to-right. The dominance of forward over reverse reads shows that replication direction is primarily right-to-left, accumulations of forward reads without a decrease in reverse reads most likely represents increased average fork residency indicative of slower fork progression or more frequent stalling, see Kara *et al.* for more details (64). TrAEL-seq profiles are an average of two biological replicates of *P_GAL1_-HA cup1* wild-type and *sac3*Δ cells grown to mid-log in 2% raffinose, with or without a 6 h 0.02% galactose induction. Reads were quantified per million reads mapped in 50 bp windows spaced every 10 bp, and an enrichment normalization applied to make overall read count distributions as uniform as possible, see Supplementary Figure S3B for the distribution of reads across single copy regions of chromosome VIII. Regions of significant difference between wild type and *sac3*Δ in 0.02% galactose were called using edgeR (65), with forward reads quantified in 200 bp windows spaced every 50 bp, no normalization was applied prior to the Edge algorithm. (**D**) Plots of TrAEL-seq read density on forward and reverse strands across a *P_GAL1_-HA cup1* repeat for wild-type cells induced for 6 h with 0%, 0.02% or 2% galactose. TrAEL-seq profiles are an average of two biological replicates, 0% and 0.02% conditions are the same data as (C), data is processed as in (C).

We recently developed TrAEL-seq to detect replication fork stalling and replication intermediates (64). Unfortunately, TrAEL-seq is ineffective for copper-treated cells as copper-induced apoptotic fragments generate a high background (64,89) (example data is deposited at GEO GSE154811). However, we have also created a *P_GAL1_-HA cup1* strain in which *GAL1* promoters replace all *CUP1* promoters, and *CUP1* ORFs are replaced by *3HA* ORFs, allowing transcriptional induction at the *cup1* locus using the non-toxic sugar galactose (Figure 3A) (49).

TrAEL-seq reads accumulate at sites of replication fork stalling, including at the endogenous *GAL1* promoter when transcriptionally active, providing a measure of disruption to replication fork progression (64). The activity of the *GAL1* promoter is exceptionally strong under normal 2% galactose induction, leading to saturating levels of Sac3- and Thp1-independent CNV in 10 generations that probably result from direct collisions between RNA polymerase and the replication fork (Supplementary Figure S3A). However, CNV is Sac3- and Thp1-dependent under moderate 0.02% galactose induction that should better reflect *CUP1* gene expression (Figure 3B), so we constructed TrAEL-seq libraries from *P_GAL1_-HA cup1* wild-type and *sac3*Δ cells induced with 0.02% galactose.

We expected to observe a pronounced accumulation of TrAEL-seq reads at the *P_GAL1_-HA* promoter as a result of replication fork stalling, but the promoter peaks formed in 0.02% galactose were modest and Sac3-independent (Figure 3C), although further enhanced in 2% galactose (Figure 3D). In contrast, the region upstream of the *P_GAL1_* promoter containing the ARS element accumulated significantly more TrAEL-seq reads in wild type than *sac3*Δ cells on induction with 0.02% galactose (right hand side and far left of Figure 3C). Normalization errors could also give rise to such a baseline signal increase, however global TrAEL-seq read distributions were unaffected by addition of galactose (Supplementary Figure S3B shows all equivalent windows in single copy regions of chromosome VIII), and if anything, the normalization process globally increases *sac3*Δ signals compared to wild-type. Therefore, we observe a small Sac3-dependent increase in TrAEL-seq read density outside the transcribed region and particularly around the ARS element in *P_GAL1_-HA cup1* suggesting that replication forks stall but not at well-defined sites.

The Sac3-dependent increase in TrAEL-seq reads across *P_GAL1_-HA cup1* is consistent with replication forks stalling due to DNA topological constraint, which would not happen at a defined location but rather happen at random across a wide area. Stalled replication forks are most often rescued by a converging replication fork, but in areas of widespread replicative stress the converging fork may be delayed and alternative replication fork restart pathways such as BIR invoked.

### *CUP1* amplification occurs in response to defective DNA polymerase δ elongation

**Figure 4.**
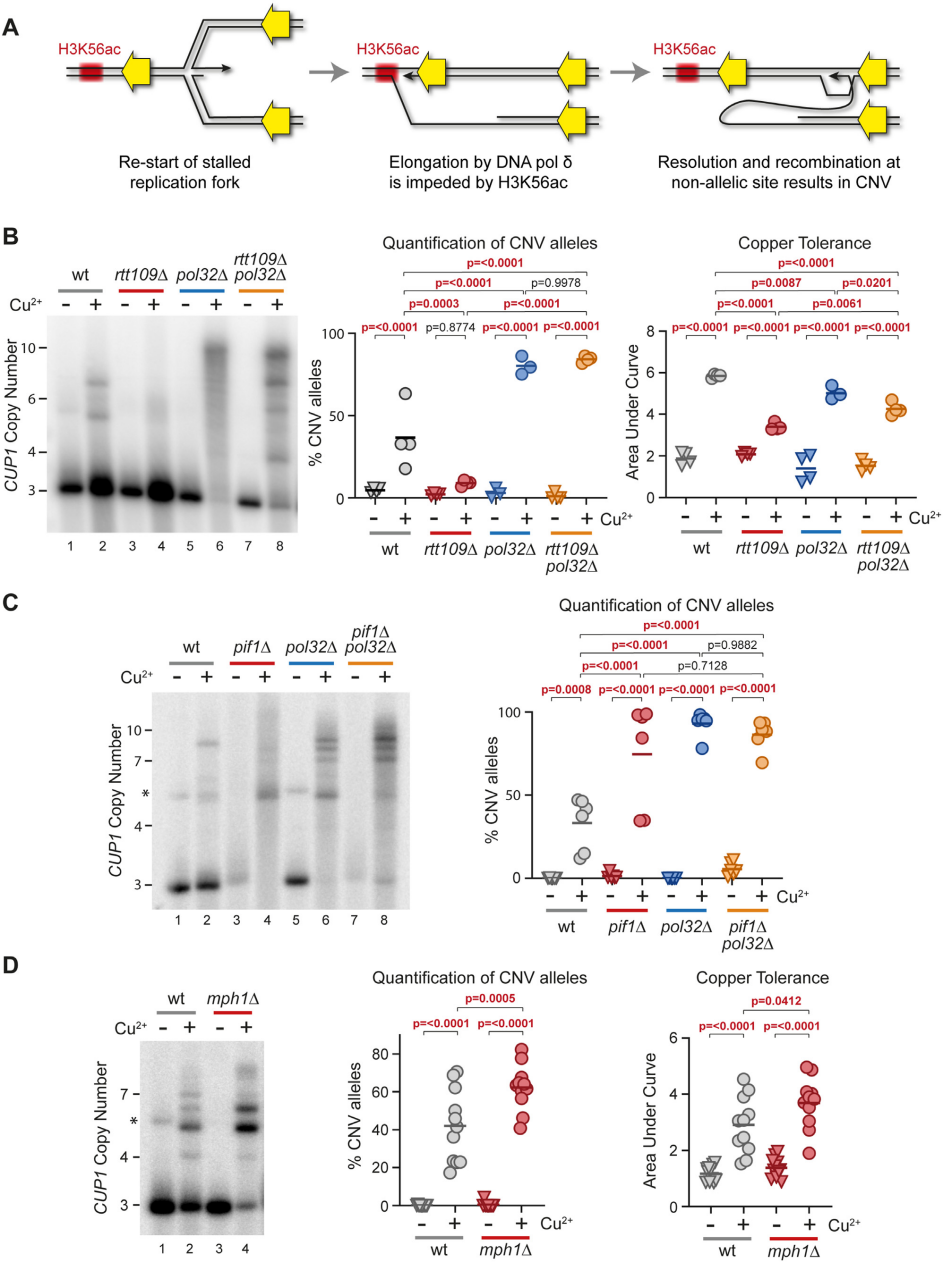
Importance of impaired DNA polymerase δ elongation in *CUP1* CNV. (**A**) Proposed mechanism leading to *CUP1* CNV. Left—a replication fork stalls and is re-started after reversal using DNA polymerase δ in a BIR-type mechanism. Middle—elongation is impaired on encounter with H3K56ac chromatin. Right—cleavage of replication fork yields a free end capable of strand invasion at a non-allelic locus, resulting in CNV. (**B**) Southern analysis of *CUP1* copy number in *3xCUP1* wild-type (wt), *rtt109*Δ, *pol32*Δ and *rtt109*Δ *pol32*Δ cells after 10 generations ±0.3 mM CuSO_4_. Quantification shows the percentage of alleles deviating from the parental copy number, *P*-values calculated by one-way ANOVA. Copper adaptation was assessed by treated cells with varying concentrations of CuSO_4_ and incubating for 3 days. Final OD_600_ was plotted against [CuSO_4_] and copper tolerance quantified as area-under-curve for each culture; *P*-values were calculated by one-way ANOVA; *n* = 4. (**C**) Southern blot analysis of *CUP1* copy number in *3xCUP1* wild-type (wt), *pif1*Δ, *pol32*Δ and *pif1*Δ *pol32*Δ cells after 10 generations ±0.3 mM CuSO_4_, analysed as in (B), *n* = 6. Asterisk indicates non-specific band in some non-copper treated samples migrating just above the 5x*CUP1* mark; this band is occasionally visible and likely represents a rare rearranged form of the locus. It does not bestow altered copper resistance and is never observed in the copper treated samples, and is therefore excluded from the quantification. (**D**) Southern blot analysis of *CUP1* copy number and adaptation test in *3xCUP1* wild-type (wt) versus *mph1*Δ cells after 10 generations ±0.3 mM CuSO_4_, analysed as in (B), *n* = 11. Asterisk indicates non-specific band, see (C) for details.

We previously proposed a model for *CUP1* CNV in which BIR-type events are initiated at the *CUP1* locus to resolve stalled replication forks, but are impeded on encountering H3K56ac chromatin, leading to secondary non-allelic recombination events that result in CNV (illustrated in Figure 4A) (49). This mechanism explains two key genetic dependencies of transcriptionally stimulated CNV at *CUP1*: the absolute dependence of CNV on the H3K56 acetyltransferase Rtt109, and the dramatic enhancement of CNV observed in cells lacking Pol32. The genetic screen confirmed the importance of H3K56ac through the impact of H3K56Q and R mutations (Figure 1C) and identified recombination factors important for *CUP1* CNV (Rad52, Rad59), but certain factors had much less effect than we expected (Rad51, Mus81) (Figure 1C, Supplementary Table S3) leading us to probe the recombination mechanism in more detail.

In this mechanism, impairment of DNA polymerase δ elongation at H3K56ac chromatin is the critical step leading to CNV. If H3K56ac is absent then elongation should proceed until eventual resolution by an oncoming replication fork, but mutations that impair DNA polymerase δ elongation should induce CNV irrespective of H3K56ac. To test this, we combined *rtt109*Δ, which abrogates H3K56ac, with *pol32*Δ, which impairs BIR elongation. *rtt109*Δ completely suppressed CNV and copper adaptation as we have previously shown (49) (Figure 4B, lanes 1–4), whereas *pol32*Δ significantly enhanced CNV (Figure 4B, lanes 5–6). Importantly, the double *rtt109*Δ *pol32*Δ mutant underwent high levels of CNV equivalent to the *pol32*Δ single mutant (Figure 4B, lanes 7–8), consistent with the idea that H3K56ac causes CNV by impairing elongation.

A specific requirement for an inefficient BIR-type mechanism is unusual and Pol32-dependence alone is open to other interpretations. We therefore examined the helicase Pif1, which is also critical for BIR elongation, and observed that deletion of *PIF1* both alone and in combination with *pol32*Δ increased amplification of the 3x*CUP1* allele compared to wild type (Figure 4C). Unfortunately, slow growth and extensive flocculation prevented determination of copper resistance in *pif1*Δ cells, but the gene amplification phenotype is consistent with the hypothesis that defects in DNA polymerase δ elongation promote *CUP1* CNV.

Whereas Pif1 and Pol32 are required for elongation in BIR-type mechanisms, the helicase Mph1 suppresses BIR through unwinding duplexes formed by strand invasion (90,91). The mechanism shown in Figure 4A requires efficient strand invasion both to initiate synthesis by DNA polymerase δ and to facilitate second strand invasion leading to CNV. This requirement is validated by the phenotype of *mph1*Δ mutants, which underwent increased *CUP1* CNV and adaptation in the 3x*CUP1* background (Figure 4D).

The phenotypic impacts of *pol32*Δ, *pif1*Δ and *mph1*Δ on CNV provide strong support for the hypothesis that *CUP1* CNV arises through a BIR-type mechanism. However, it is not efficient processive elongation after fork restart that leads to CNV, but rather situations in which elongation is impeded by chromatin structure or genetic defects.

### Requirement for structure specific endonucleases and recombination proteins in *CUP1* CNV

Once DNA polymerase δ elongation is impeded, the replication fork can be resolved either by an oncoming replication fork, which would maintain parental copy number, or by structure specific endonucleases (SSEs). In a nuclease-induced DSB model, resolution of BIR intermediates by an SSE results in a half-crossover product containing a free DNA end that could initiate strand invasion events at non-allelic sites (23,24).

**Figure 5.**
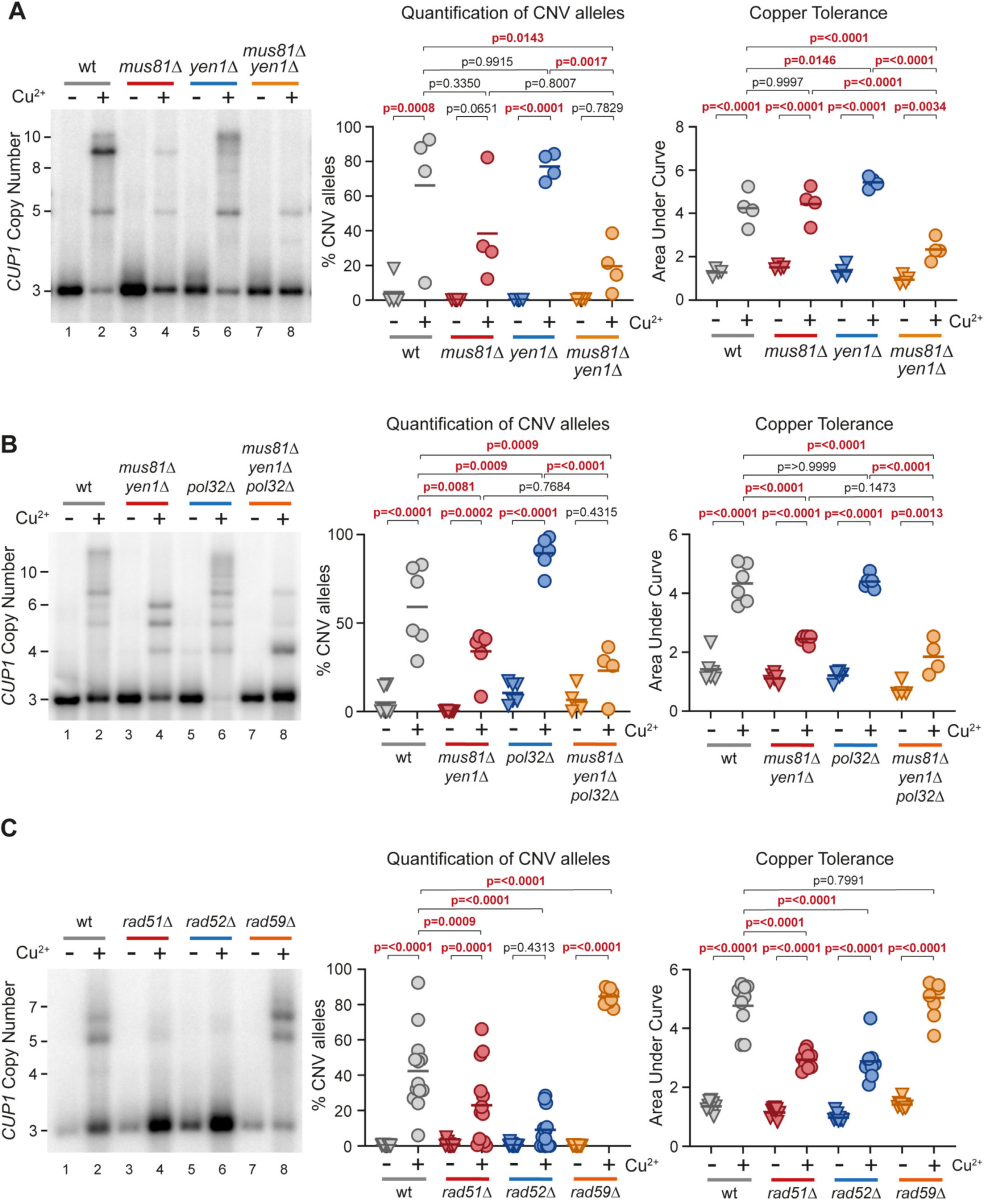
Importance of SSEs and Rad proteins in *CUP1* CNV. (**A**) Southern analysis of *CUP1* copy number in *3xCUP1* wild-type (wt), *mus81*Δ, *yen1*Δ and *mus81*Δ *yen1*Δ cells after 10 generations ±0.3 mM CuSO_4_. Quantification shows the percentage of alleles deviating from the parental copy number, *P*-values calculated by one-way ANOVA. Copper adaptation was assessed by treated cells with varying concentrations of CuSO_4_ and incubating for 3 days. Final OD_600_ was plotted against [CuSO_4_] and copper tolerance quantified as area-under-curve for each culture; *P*-values were calculated by one-way ANOVA; *n* = 4. (**B**) Southern blot analysis of *CUP1* copy number and adaptation test in *3xCUP1* wild-type (wt), *mus81*Δ *yen1*Δ, *pol32*Δ and *mus81*Δ *yen1*Δ *pol32*Δ cells after 10 generations ±0.3 mM CuSO_4_, analysed as in (A), *n* = 6. (**C**) Southern blot analysis of *CUP1* copy number and adaptation test in *3xCUP1* wild-type (wt), *rad51*Δ, *rad52*Δ and *rad59*Δ cells after 10 generations ±0.3 mM CuSO_4_, analysed as in (A), *n* = 12.

Three SSE complexes with overlapping substrate specificities have been characterized in budding yeast—Mus81–Mms4, Yen1 and Slx1–Slx4 (reviewed in (92)). *mus81*Δ and *yen1*Δ mutations individually had no effect in the genetic screen and little effect on *CUP1* amplification in the 3x*CUP1* background (Figure 5A, lanes 3–6), however, the double mutant *mus81*Δ *yen1*Δ displayed a robust CNV suppression phenotype and reduced copper adaptation (Figure 5A, lanes 7–8). Additional deletion of *SLX4* did not further reduce *CUP1* amplification or adaptation in a *mus81*Δ *yen1*Δ background (Supplementary Figure S4A), so therefore Mus81 and Yen1 act redundantly to provide SSE activity required for efficient *CUP1* amplification.

Constitutive impairment of elongation in *pol32*Δ overcomes the CNV suppression caused by *rtt109*Δ, but SSE activity should be important for resolution irrespective of whether elongation is impeded by H3K56ac chromatin or lack of Pol32. As predicted, deletion of *POL32* did not restore *CUP1* CNV or adaptation in 3x*CUP1 mus81*Δ *yen1*Δ, showing that SSE activity is an intrinsic and critical step in the mechanism (Figure 5B).

SSE-mediated resolution creates a free DNA end that is predicted to be highly recombinogenic. We expected that secondary strand invasion events instigated by the free DNA end would be mediated by Rad52 and Rad51, but *rad51*Δ had no effect in the genetic screen whereas *rad59*Δ abrogated CNV. We therefore analysed these mutants in the 3x*CUP1* system. In contrast to the genetic screen, which measures only contraction of the *CUP1* array, *CUP1* amplification and copper adaptation in 3x*CUP1* were strongly reduced in *rad51*Δ and almost absent in *rad52*Δ (Figure 5C, lanes 1–6), whereas CNV significantly increased in *rad59*Δ (Figure 5C lanes 7,8). This shows that *CUP1* copy number amplifications and contractions are mediated by separate mechanisms that act in competition: a Rad59-mediated pathway causes the majority of contraction events, as previously observed (93), but amplifications depend on Rad51, and some DNA ends that would be processed to contractions by Rad59 in the wild type are diverted to Rad51-mediated amplifications in *rad59*Δ.

These data are consistent with a model in which Mus81 or Yen1 cleave the replication fork after DNA polymerase δ is impeded, and the resulting free DNA end is resected then annealed by Rad59 or Rad51 to yield *CUP1* copy number contractions or amplifications respectively.

### Replication fork progression is a critical mediator of *CUP1* CNV

Gene gating is common (78) and if each gene gating event caused sufficient replication stress to instigate error-prone BIR-type mechanisms then dramatic genome instability would ensue. This suggests that the *CUP1* locus is particularly prone to CNV, and to gain further insights into the properties of this locus we explored the potent CNV-stimulatory effect of loss of Mrc1, the strongest suppressor in the genetic screen (Figure 1F).

**Figure 6.**
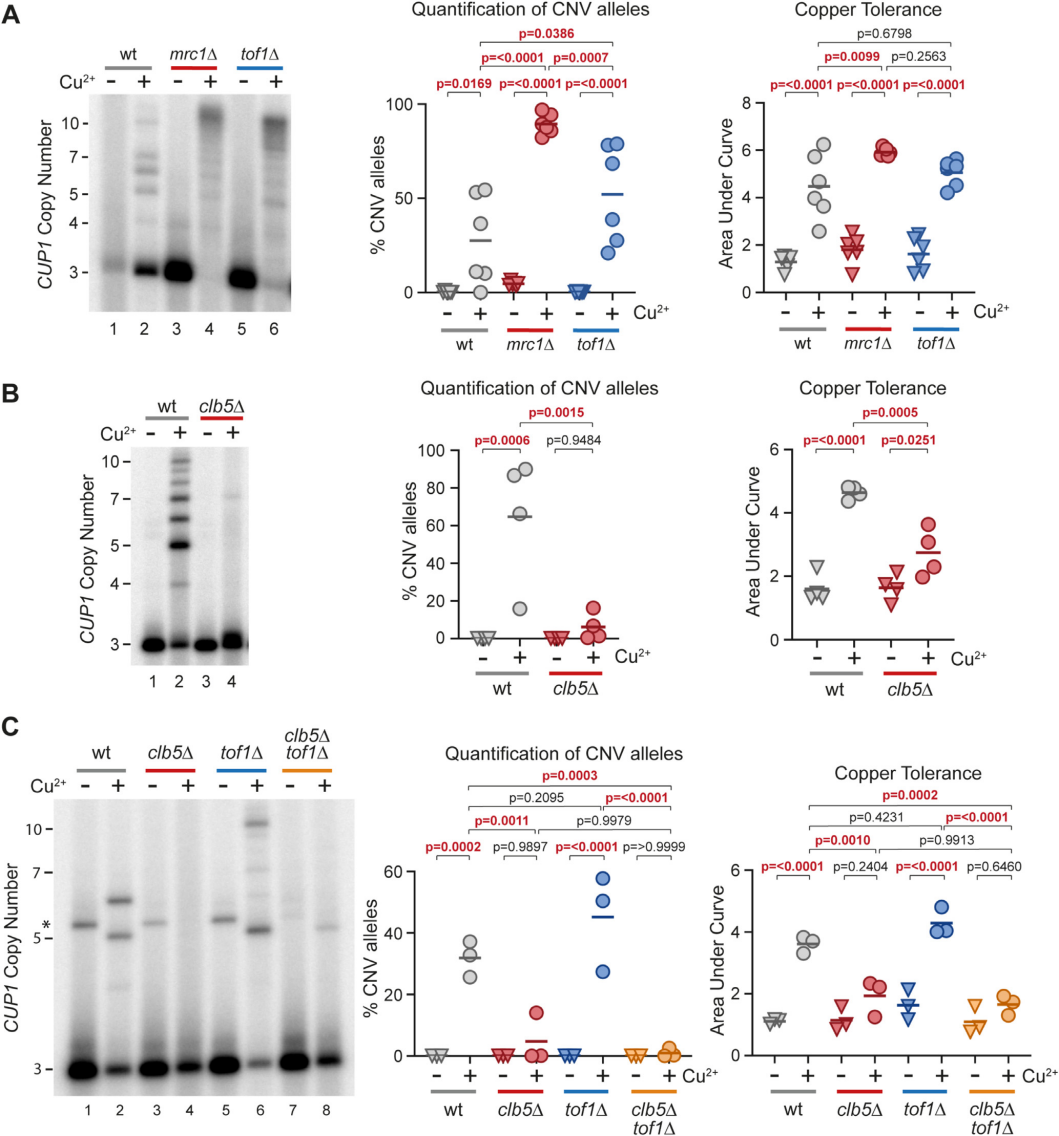
Effect of replication timing and replication fork progression on *CUP1* CNV. (**A**) Southern analysis of *CUP1* copy number in *3xCUP1* wild-type (wt), *mrc1*Δ and *tof1*Δ cells after 10 generations ±0.3 mM CuSO_4_. Quantification shows the percentage of alleles deviating from the parental copy number, *P*-values calculated by one-way ANOVA. Copper adaptation was assessed by treating cells with varying concentrations of CuSO_4_ for 3 days, final OD_600_ was plotted against [CuSO_4_] and copper tolerance quantified as area-under-curve for each culture; p-values were also calculated by one-way ANOVA; *n* = 6. (**B**) Southern blot analysis of *CUP1* copy number and adaptation test in *3xCUP1* wild type (wt) and *clb5*Δ mutants. Cells were cultured and CNV alleles and copper adaptation quantified as in (A); *n* = 4. (**C**) Southern blot analysis of *CUP1* copy number and adaptation test in *3xCUP1* wild type (wt), *clb5*Δ, *tof1*Δ and *clb5*Δ *tof1*Δ cells. Cells were cultured, CNV alleles and copper adaptation quantified as in (A); *n* = 3. Asterisk indicates non-specific band, see Figure 4C for details.

We first asked whether Mrc1 suppresses *CUP1* CNV by mediating the DNA replication checkpoint (94,95), however the absence of key replication checkpoint proteins Mec1 and Rad53 did not increase CNV or adaptation in 3x*CUP1* cells (Supplementary Figure S5A). This result was unexpected due to the involvement of Mec1 in both maintaining replication fork stability and releasing gated genes from the nuclear pore. Loss of Mec1 would therefore be predicted to increase CNV, but this may be compensated for by reduced activation in *mec1*Δ cells of the DNA repair pathways that generate CNV. Mrc1 also regulates replication fork speed in concert with Tof1 and Csm3 (96,97), and deletion of *TOF1* in 3x*CUP1* phenocopied *mrc1*Δ in significantly accelerated *CUP1* CNV, suggesting that replication fork speed is a major determinant of CNV rate (Figure 6A). Reducing replication fork speed would increase the chance that late replicating regions are not replicated prior to G2/M, requiring emergency repair by BIR-type mechanisms as at mammalian fragile sites (37) and promoting the use of inefficient late firing replication origins (98,99). The *CUP1* locus is located in a particularly late replicating region of Chromosome VIII and contains poorly characterized ARS elements (Supplementary Figure S5B and Figure 1A), so both delayed replication of the locus and activation of local replication origins are plausible contributors to CNV.

If either late replication or local origin activation is important, then altering the replication profile should impact CNV. We therefore deleted the gene encoding Clb5, a cyclin that regulates late firing replication origins; replication fork progression is normal in *clb5*Δ mutants (100) but loss of Clb5 prevents firing of late origins and S phase is extended to allow completion of replication (101,102). We observed that *CUP1* CNV decreased substantially in *clb5*Δ cells with a concurrent reduction in copper adaptation (Figure 6B), showing that Clb5 activity is important for *CUP1* CNV. To confirm this result we tested two other mutants, *sic1*Δ and *dia2*Δ, which cause similar shifts in replication and origin use to *clb5*Δ (98) albeit through a variety of mechanisms, and obtained the same result (Supplementary Figure S5C). Therefore, Clb5 activity is required for efficient *CUP1* CNV.

If *clb5*Δ suppresses CNV by extending S-phase such that the *CUP1* locus has more time to replicate, then slowing replication forks by removing Mrc1 or Tof1 should restore CNV. Unfortunately, the *mrc1*Δ *clb5*Δ double mutant was not viable in this background, however, deletion of *CLB5* in 3x*CUP1 tof1*Δ completely suppressed *CUP1* CNV and copper adaptation (Figure 6C). This is hard to reconcile with a model in which *CLB5* deletion simply extends replication timing to allow replication forks more time to finish DNA synthesis as we would not expect this to completely offset the replication fork slowing caused by *tof1*Δ, so other effects of *CLB5* deletion likely underlie the suppression of CNV.

### Late firing replication origin activity promotes *CUP1* CNV

Since extension of S-phase in *clb5*Δ and *sic1*Δ mutants is attributed to suppression of late-firing replication origins (98,102,103), and conversely deletion of *MRC1* increases usage of late firing origins (95,98), it is possible that local origin activity is the principle determinant of *CUP1* CNV, rather than replication timing or replication fork speed *per se*. Replication origins (ARS elements) are present in each of the *CUP1* repeats although whether these are active during S phase under normal conditions is unclear (Figure 1A).

**Figure 7.**
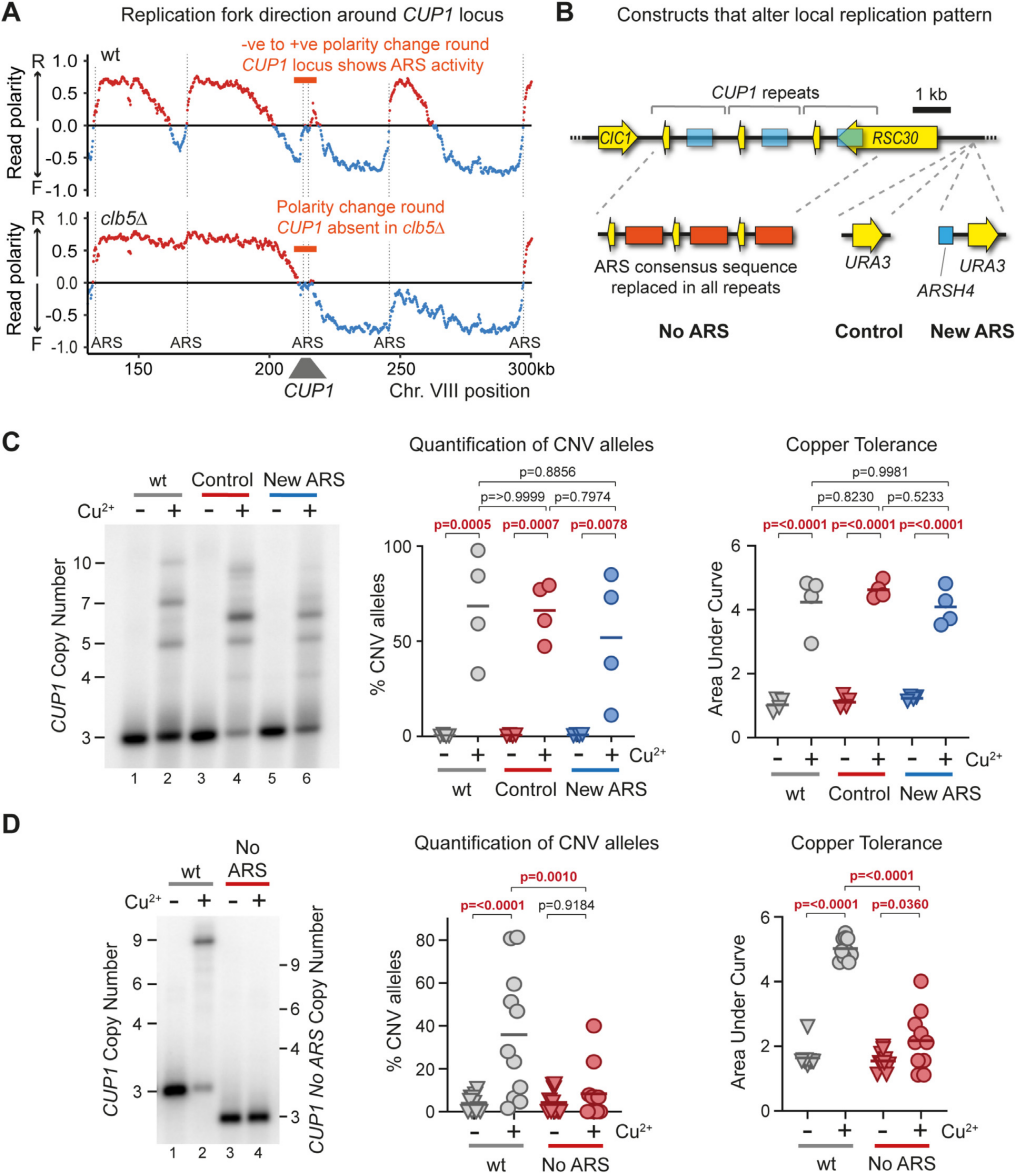
Local Replication origin firing regulates CNV at *CUP1* locus. (**A**) TrAEL-seq read polarity plots for wild type and *clb5*Δ cells grown to mid log in 2% Glucose. TrAEL-seq detects replication direction, an excess of reverse (R) reads at any site (indicated by positive polarity, red), results from replication forks moving left-to-right, the opposite for replication forks moving right-to-left (blue). Sharp transitions from –ve to +ve occur at active replication origins (ARS elements), gradual transitions from +ve to –ve are regions where forks converge. Plots show regions surrounding *CUP1* on chromosome VIII, quantified by (R-F)/(R + F) with R and F referring to Reverse and Forward reads respectively; *n* = 2, data from GSE154811. (**B**) Schematic of the three copy *CUP1* array and the modifications introduced to influence local replication pattern. In ‘No ARS’ cells, the region containing the endogenous ARS upstream of every *CUP1* repeat have been replaced by a non-expressed sequence derived from a *GFP* tagging plasmid. ‘New ARS’ and ‘Control’ cells have a *URA3* marker integrated upstream of the *RSC30* promoter, either with or without an efficient replication origin (*ARSH4*) respectively. TrAEL-seq data confirming activity of the new ARS is shown in Supplementary Figure S5A. (**C**) Southern blot analysis of *CUP1* copy number in *3xCUP1* wild-type (wt), Control and New ARS cells (described in B). Cells were cultured for 10 generations ±0.3 mM CuSO_4_. Quantification shows the percentage of alleles deviating from the parental copy number, *P*-values calculated by one-way ANOVA. Copper adaptation was assessed by treating cells with varying concentrations of CuSO_4_ and incubating for 3 days, final OD_600_ was plotted against [CuSO_4_] and copper tolerance quantified as area-under-curve for each culture; p-values were also calculated by one-way ANOVA; *n* = 4. (**D**) Southern blot analysis of *CUP1* copy number and growth curve analysis in *3xCUP1* wild-type (wt) and *3xCUP1* cells with the region containing the ARS sites replaced by unrelated sequence (described in B). Cells were cultured, CNV alleles and copper adaptation quantified as in (B), *n* = 10 for Southern blot analysis, *n* = 11 for growth curve analysis.

Formation of a replication bubble at a replication origin creates two replication forks that move in opposite directions, and replication origins are therefore detectable as sites of sharp changes in replication fork direction. Replication fork direction is revealed by the polarity of TrAEL-seq reads (64), and TrAEL-seq data from BY4741 wild-type cells with 13 copies of *CUP1* shows a change in polarity from negative to positive at the *CUP1* origin indicative of ARS activity (Figure 7A upper panel, highlighted in orange). This signal is weak compared to other ARS elements in the region and becomes almost undetectable in 3x*CUP1* cells (Supplementary Figure S6A, upper panel), consistent with the *CUP1* ARS elements being functional but rarely active. Very little change in replication fork polarity is detected at *CUP1* in *clb5*Δ cells with 13 copies of *CUP1*, placing the *CUP1* ARS elements amongst those origins dependent on Clb5 (Figure 7A lower panel), whereas TrAEL-seq of 3x*CUP1 mrc1*Δ reveals pronounced replication origin activity at *CUP1* that is not detected in the 3x*CUP1* wild type, consistent with increased usage of late-acting replication origins (Supplementary Figure S6A lower panel) (95,98).

The presence of Clb5-dependent ARS elements at *CUP1* is therefore consistent with the possibility that *CUP1* ARS activity promotes *CUP1* CNV. If so then loss of Clb5 would suppress CNV by preventing local ARS activity rather than by extending S phase. To distinguish these possibilities, we created origin deletion and insertion strains that either prevent *CUP1* ARS activity or force early replication (Figure 7B).

Firstly, to promote early replication of *CUP1* we integrated the efficient and early *ARSH4* origin adjacent to the *CUP1* locus to create a ‘New ARS’ strain, along with a control strain containing the selectable marker but no ARS element (Figure 7B). TrAEL-seq analysis confirmed that this origin is efficient and dominates the local replication profile (Supplementary Figure S6B). However, this had no detectable impact on copper adaptation or *CUP1* repeat amplification, and repeat amplification remained sensitive to *clb5*Δ (Figure 7C and Supplementary Figure S6C). Therefore, *CUP1* amplification does not depend on replication forks arriving at the locus late in S-phase from distant origins, and the extension of S-phase in *clb5*Δ cannot explain the strong suppression of CNV.

Secondly, to prevent replication origin firing at *CUP1* we mutated the ARS element in each *CUP1* repeat. As there are considerable discrepancies in the locations assigned to the *CUP1* ARS element (104,105) we deleted a substantial region of each *CUP1* repeat upstream of the *CUP1* promoter, replacing this with unrelated sequence derived from a *GFP* construct (Figure 7B, ‘no ARS’). This change did not alter basal copper resistance compared to 3x*CUP1* cells containing wild type *CUP1* repeats (Supplementary Figure S6D, top panel), confirming that *CUP1* function was unaffected. However, when pre-exposed to copper, loss of the ARS suppressed *CUP1* CNV and copper adaptation to a similar extent as *CLB5* deletion (Figure 7D). This shows that activity of local ARS elements in the *CUP1* repeats, which is suppressed in *clb5*Δ, drives *CUP1* CNV. Interestingly, efficient *CUP1* amplification was restored in the No ARS strain when H3K56ac was rendered constitutive by removal of the H3K56 deacetylases Hst3 and Hst4, indicating that *CUP1* ARS activity contributes to CNV primarily through deposition of H3K56ac, although the growth defect caused by *hst3*Δ *hst4*Δ meant that we could not determine whether this also restored adaptation (Supplementary Figure S6E).

These experiments resolve the contribution of Clb5 and Mrc1 to the *CUP1* CNV phenotype, showing that activation of inefficient replication origins at *CUP1* rather than the late replication timing of the locus underlies the replication-dependence of transcriptionally stimulated *CUP1* amplification. Taken together with our previous results, this suggests that replication origin activation, in regions of high topological strain caused by promoter-nuclear pore interactions, leads to increased CNV through an inefficient BIR-type mechanism.

## DISCUSSION

Here, we have dissected the mechanism by which transcriptional induction of the copper resistance gene *CUP1* stimulates CNV events that cause *CUP1* amplification and thereby increase copper resistance. We demonstrate critical roles for the TREX-2 and Mediator complexes that link transcribed loci to the nuclear pore, and for local replication origin activity, in addition to the known importance of H3K56ac.

### A two-step mechanism for *CUP1* CNV

Given recent discoveries of replication fork instability resulting from collisions between replication forks and RNA polymerase II or R-loops, the results of our genetic screen for *CUP1* CNV modulators were not as we expected (44,45,54,55,58). The importance of TREX-2 and Mediator, deletions of which are even stronger suppressors of copper adaptation than *rad52*Δ, suggests that topological constraint of replication fork progression causes CNV, rather than direct interactions with the transcription unit, and this is coherent with the small but widespread increase in TrAEL-seq read density when the locus is transcriptionally active. Nonetheless, topological impairment of replication fork progression is known (56,57) and it is not too surprising that this increases the frequency of homologous recombination events that can result in CNV.

However, the requirement for H3K56ac needs to be explained (49). H3K56ac impairs DNA polymerase δ elongation during BIR (43), but given that H3K56ac should only be present on new histones that are deposited behind the replication fork, it is unclear why DNA polymerase δ encounters H3K56ac during replication fork re-start as the histones ahead of the replication fork should not carry H3K56ac (106,107). One possibility is that abortive replication origin firing deposits H3K56ac: if both forks in the nascent replication bubble stall due to high topological strain then the replication bubble could dissolve by fork reversal and/or nascent strand degradation (illustrated in Supplementary Figure S7A). During the formation and initial progression of the bubble, histone exchange would leave an ‘epigenetic scar’ of H3K56ac chromatin in this non-replicated genomic region (also illustrated in Supplementary Figure S7A). We acknowledge that this would require a very pronounced fork reversal or degradation, but the restoration of adaptive *CUP1* CNV in the no ARS strain by deletion of *HST3* and *HST4*, which causes H3K56ac to be present throughout the genome (108), does suggest that the *CUP1* origins facilitate CNV through H3K56ac deposition.

**Figure 8.**
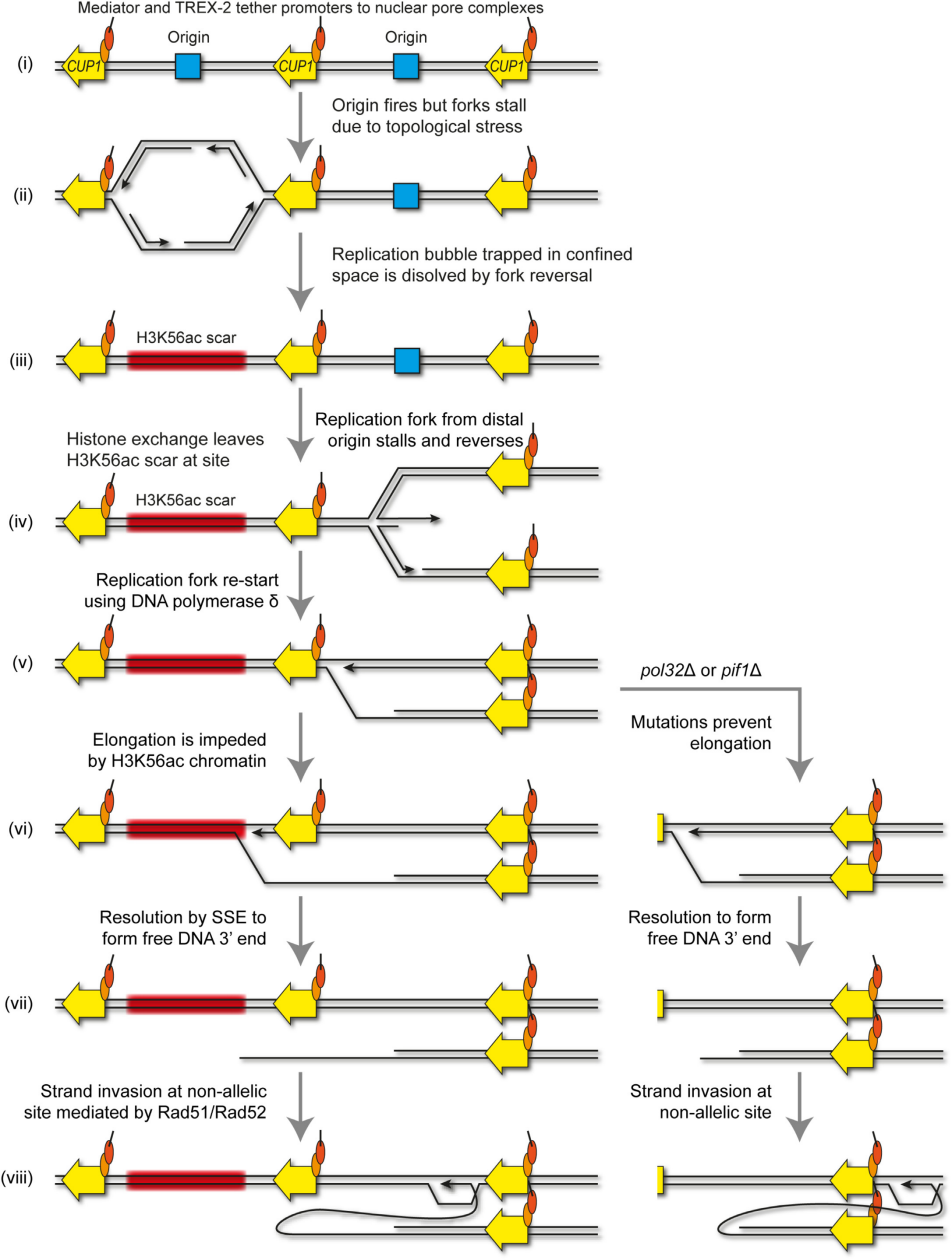
A model for origin-dependant stimulated-CNV of *CUP1* locus. DNA strands are shown in black, the *CUP1* gene shown in yellow, replication origins are shown in blue, chromatin containing H3K56ac shown in red, and mediator and TREX-2 complexes are shown as orange and red circles respectively. See Discussion for more details. Replication fork re-start is represented following the model described in (33), resulting in elongation by DNA polymerase δ without concurrent Okazaki fragment synthesis. The elongating re-started fork or the new fork formed by strand invasion will eventually be resolved by a replication fork converging from the left (not shown), allowing semi-conservative synthesis replication of the lagging strand by DNA polymerase δ (32). The slightly more complex outcome, Rad59-mediated resolution with a reversed converging fork, is illustrated in Supplementary Figure S7B.

The overall mechanism we propose for transcriptionally-stimulated *CUP1* CNV is illustrated in Figure 8. Firstly, replication is initiated from a replication origin in the *CUP1* region but rapidly stalls and the replication bubble collapses due to replicative stress imposed by TREX-2 and Mediator, leaving a tract of H3K56ac chromatin in an un-replicated genomic region (Figure 8, steps i–iii). A second replication fork arriving from a distal site stalls due to local replicative stress and initiates fork re-start using DNA polymerase δ through fork reversal and strand invasion (Figure 8, steps iv–v), but elongation is impaired on encounter with the H3K56ac chromatin (Figure 8, step vi). Resolution by a SSE releases a free DNA end (Figure 8, step vii) that can undergo Rad51-mediated strand invasion with the sister chromatid to yield an amplification or a contraction if invasion occurs at a non-allelic site (Figure 8, step viii). If H3K56ac has not been deposited ahead of the replication fork that arrives in step iv (as in *rtt109*Δ), the stalling and re-start process would still occur, but the SSE resolution and non-allelic strand invasion steps (vi–viii) that result in CNV are avoided. Irrespective of any impediments to elongation and secondary strand invasion events, resolution by a converging replication fork (coming from the left in Figure 8) is finally required to complete chromosomal replication following step viii.

In *pol32*Δ and *pif1*Δ mutants with defective DNA polymerase δ elongation, SSE resolution must occur close to the site of re-start, but the outcome is otherwise the same as a free DNA end that can undergo non-allelic strand invasion is again formed (Figure 8 alternate pathway steps vi–viii). This event is however no longer dependent on H3K56ac, and is probably more efficient than in wild-type as it seems unlikely that H3K56ac inhibits DNA polymerase δ elongation as effectively as the absence of Pif1 or Pol32. It should be noted that this requirement for resolution by an oncoming fork, which is common in repair of stalled forks by BIR-type mechanisms (38–40), means that the BIR fork does not need to elongate any significant distance, and in *pol32*Δ or *pif1*Δ mutants the SSE resolution and strand invasion (steps vii and viii) would occur immediately following the initial strand invasion, and even if the intermediate in step viii is not elongated, resolution by an oncoming fork would still result in amplification.

An alternative pathway at step viii would be single strand annealing of the free DNA end with the oncoming fork, which results in a contraction (Supplementary Figure S7B). The high efficiency of single strand annealing compared to strand invasion probably explains the bias of *CUP1* CNV towards contractions that we have previously noted (49,93,109), but the Rad51-mediated pathway still yields sufficient amplification events to reproducibly emerge under the mild copper selection applied in the 3x*CUP1* adaptation system (Figure 1E). Therefore, transcriptionally stimulated CNV at the *CUP1* locus emerges through at least two competing pathways with partially overlapping outcomes. The mechanism by which CNV emerges, recombination resulting from elongation difficulties after replication fork re-start, is conceptually similar to the microhomology-mediated BIR (MMBIR) model, proposed to explain complex rearrangements in human cells (11). However, recombination at *CUP1* is strictly homology dependent rather than utilizing microhomology, and occurs in response to a defined chromatin signature that is at present unknown in MMBIR.

### Replication stress as a driver of adaptation

Adaptive mutation at *CUP1* does not fit classical models of adaptation through selection of random pre-existing mutations, as *CUP1* CNV occurs in response to copper stress. However, neither does this constitute a stress-induced mutational pathway equivalent to the bacterial SOS response or recent reports of adaptive mutability during chemotherapy (110–113). Stress induced mutation pathways invoke a switch to the use of mutagenic repair pathways for repair of random DNA damage (reviewed in (114)), whereas the frequency and location of DNA damage is dramatically increased by the replication conflicts with a highly transcribed *CUP1* allele, driving CNV without necessitating a change in repair mechanism.

More generally, we suggest that DNA replication under environmental challenge is inherently mutagenic and prone to increase the genetic heterogeneity of a population from which adaptive mutations can be selected. Cells exposed to imperfect environments may be metabolically suboptimal and have reduced levels of dNTPs, ATP or histones needed for replication, may strongly induce stress responsive genes orientated head-on to replication forks (as in bacteria (115)) and may have increased levels of DNA lesions that impair replication fork progression. These would reduce fork progression and increase the frequency of local repair events, but also incur the widespread usage of inefficient origins that we find to be mutagenic at *CUP1* and increase the likelihood of fragile sites not completing replication at G2/M (37,116–121). Therefore, increased mutation rate does not need to be actively stimulated or initiated by cells under environmental challenge as long as some level of replication is maintained, since increased genetic heterogeneity should be an emergent property of replication under stress.

The question of when and how resistance mutations form is critical; as yeast pathogens become resistant to fungicides we will face major challenges in medicine and food production (reviewed in (122)). An example of the damage wrought by fungal pathogens is the progressive annihilation of amphibians by *Batrachochytrium dendrobatidis* (reviewed in (123)). Here, we provide proof of principle that the emergence of adaptive mutations is not inevitable as multiple genetic manipulations prevent budding yeast acquiring resistance to the agricultural fungicide copper sulfate. If adaptive mutations generally arise during fungicidal treatment in medicine or agriculture then a window of opportunity for avoiding resistance exists: by altering or supplementing treatment regimens to prevent replication or diminish replicative stress, the occurrence of *de novo* mutation should be reduced.

## DATA AVAILABILITY

Sequencing data is available on GEO, accession number: GSE165163. Code for read processing is available on GitHub: https://github.com/FelixKrueger/TrAEL-seq and https://github.com/FelixKrueger/TrimGalore.

## Supplementary Material

gkab1257_Supplemental_Files

## Notes

Present address: Michelle King, Department of Biomedical Science, University of Sheffield, Sheffield, UK.

Present address: Ryan M. Hull, SciLifeLab, Department of Microbiology, Tumor and Cell Biology, Karolinska Institutet, Solna, Sweden.
