# Supplementary material for: Stimulation of adaptive gene amplification by origin firing under replication fork constraint
Source: Nucleic Acids Res. 2022 Jan 8;50(2):915–36. doi: 10.1093/nar/gkab1257 (PMC8789084; doi:10.1093/nar/gkab1257)
Supplement: gkab1257_Supplemental_Files [file gkab1257_supplemental_files.zip › Supplementary Material - revised.pdf]

Supplementary Material

Supplementary Figures

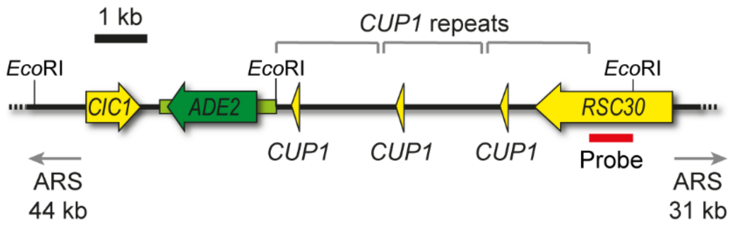

Parental *Eco*RI fragment is 6874 bp

+1 *CUP1* repeat is 8890 bp

+2 *CUP1* repeats is 10906 bp

...etc

Supplementary Figure S1: Schematic of the *CUP1* locus in the 3x*CUP1* strain. The 3x*CUP1* cassette is inserted with an *ADE2* marker containing an additional *Eco*RI site. The probe is to the non-repetitive region to ensure that hybridisation signals do not change with copy number. Each *CUP1* repeat is almost exactly 2 kb.

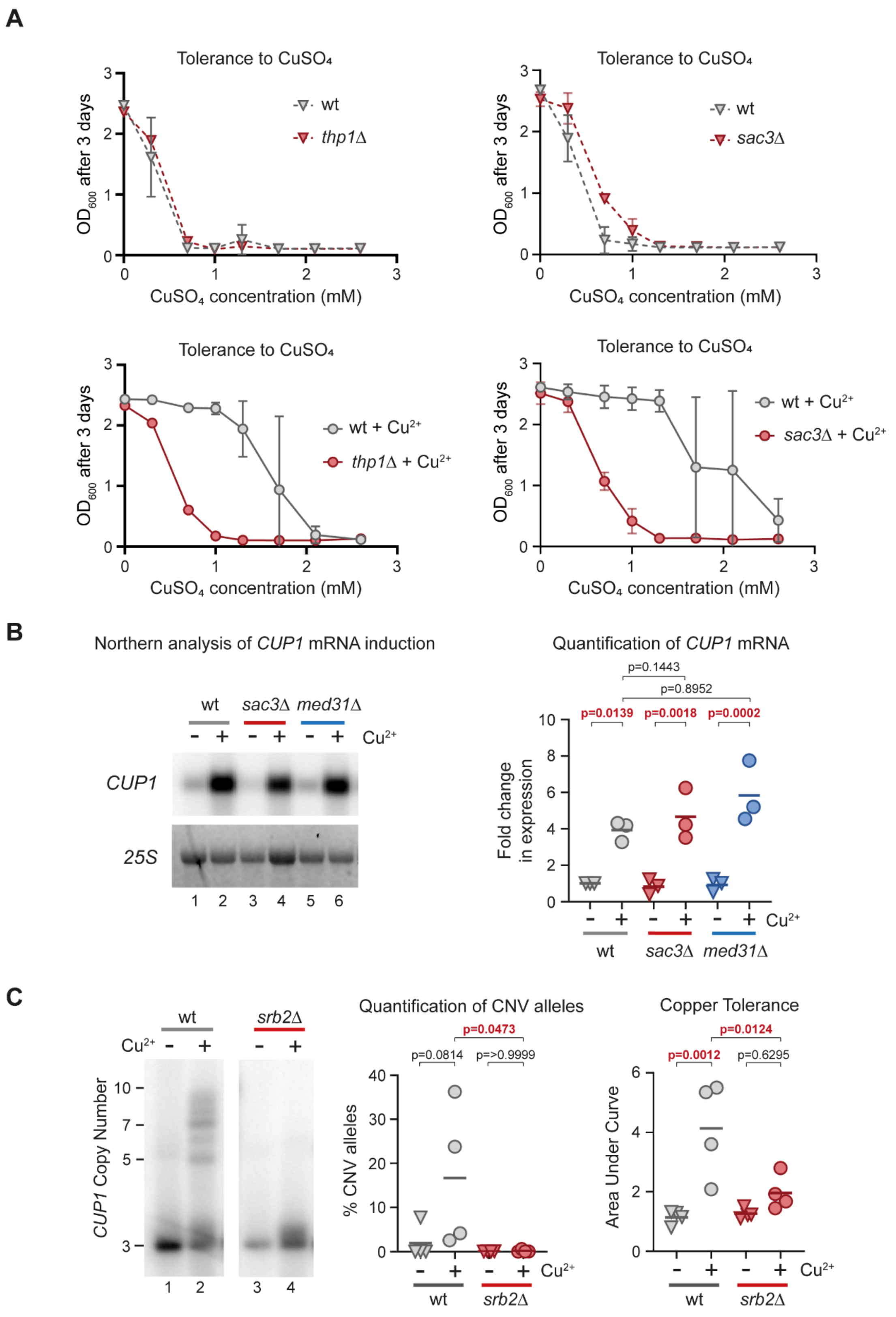
A
Tolerance to CuSO₄
OD600 after 3 days
CuSO₄ concentration (mM)
wt
thp1Δ
sac3Δ
wt + Cu2+
thp1Δ + Cu2+
sac3Δ + Cu2+
B
Northern analysis of CUP1 mRNA induction
wt sac3Δ med31Δ
Cu2+
CUP1
25S
1 2 3 4 5 6
Quantification of CUP1 mRNA
p=0.1443
p=0.8952
p=0.0139 p=0.0018 p=0.0002
Fold change in expression
wt sac3Δ med31Δ
C
wt srb2Δ
Cu2+
CUP1 Copy Number
10
7
5
3
1 2 3 4
Quantification of CNV alleles
p=0.0473
p=0.0814 p=>0.9999
% CNV alleles
Copper Tolerance
p=0.0124
p=0.0012 p=0.6295
Area Under Curve

Supplementary Figure S2. Supplement to TREX-2 and Mediator are required for transcription stimulated *CUP1* CNV. **(A)** Plots of final OD_{660} versus [$CuSO_4$] for wild type, *thp1*Δ and *sac3*Δ. Upper plots show naïve cells that have not been pre-exposed to $CuSO_4$, lower plots show cells that have been pre-cultured in 0.3mM $CuSO_4$. Tolerance of mutants that have not been pre-cultured in $CuSO_4$ is similar or higher than wild type, showing that loss of Thp1 and Sac3 does not impair the normal response to environmental copper. This is the source data underlying the plots in Figures 2B, 2C. **(B)** Northern blot analysis of *CUP1* mRNA, in wild-type, *sac3*Δ and *med31*Δ cells. Mid-log cells were treated with 0.3mM $CuSO_4$ for 6 hours and fold change in *CUP1* expression was quantified relative to the *25S* rRNA highlighted by ethidium bromide staining, p-values calculated by 1-way ANOVA; n = 3. **(C)** Southern blot analysis of *CUP1* copy number and copper tolerance analysis of *3xCUP1* wild-type (wt) and *srb2*Δ cells, performed as in Figure 2A, n = 3.

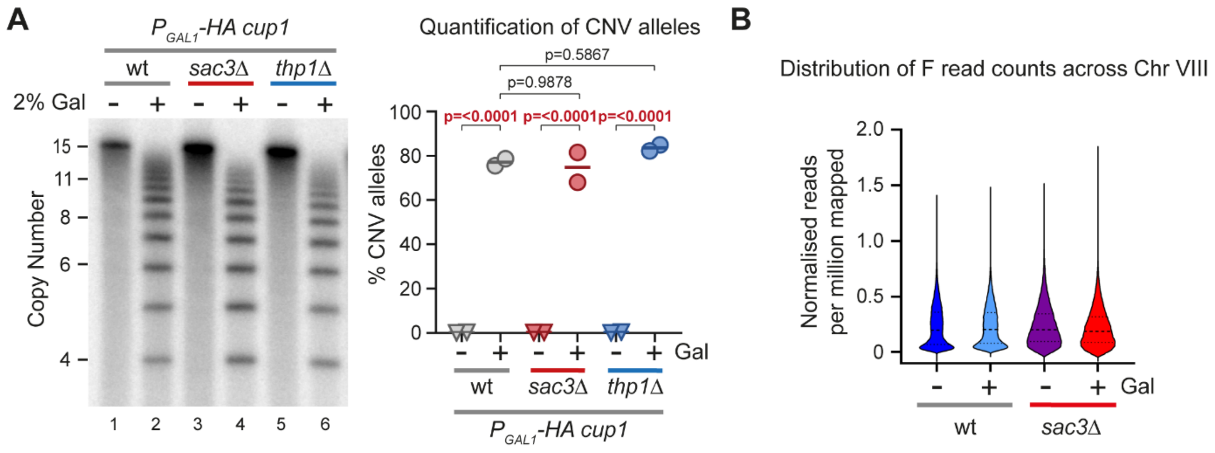

Supplementary Figure S3. Supplement to replication fork stalling and cleavage at *CUP1* locus. **(A)** Cells with every *CUP1* ORF and promoter in each *CUP1* copy replaced by P_{GAL1}-*HA*, grown for 10 generations in raffinose ± 2% galactose. Analysis by Southern blot comparing *sac3*Δ and *thp1*Δ cells to wild type. Quantification shows the percentage of alleles deviating from the parental copy number of 17 copies; n = 2. **(B)** Violin plots showing the distribution of read counts for all 50 bp windows spaced at 10 bp intervals across chromosome VIII that do not overlap multi-copy elements, data was processed as in Figure 3C.

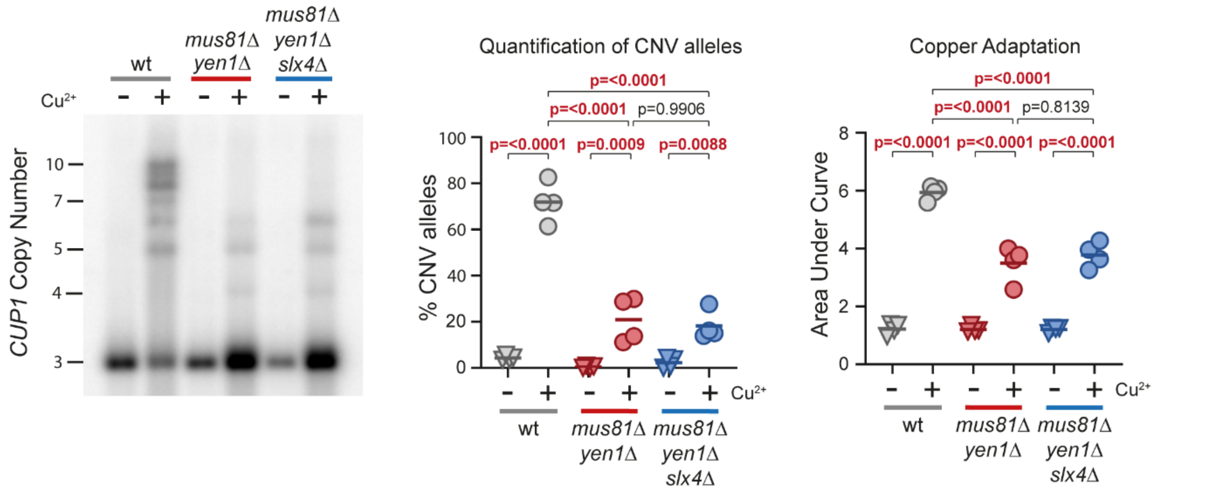

Supplementary Figure S4. Supplement to importance of SSEs and Rad proteins in *CUP1* CNV. Southern blot analysis of *CUP1* copy number and adaptation test in *3xCUP1* wild-type (wt), *mus81*Δ *yen1*Δ, and *mus81*Δ *yen1*Δ *slx4*Δ cells after 10 generations ± 0.3 mM $CuSO_4$, analysed as in Figure 5A, n=4.

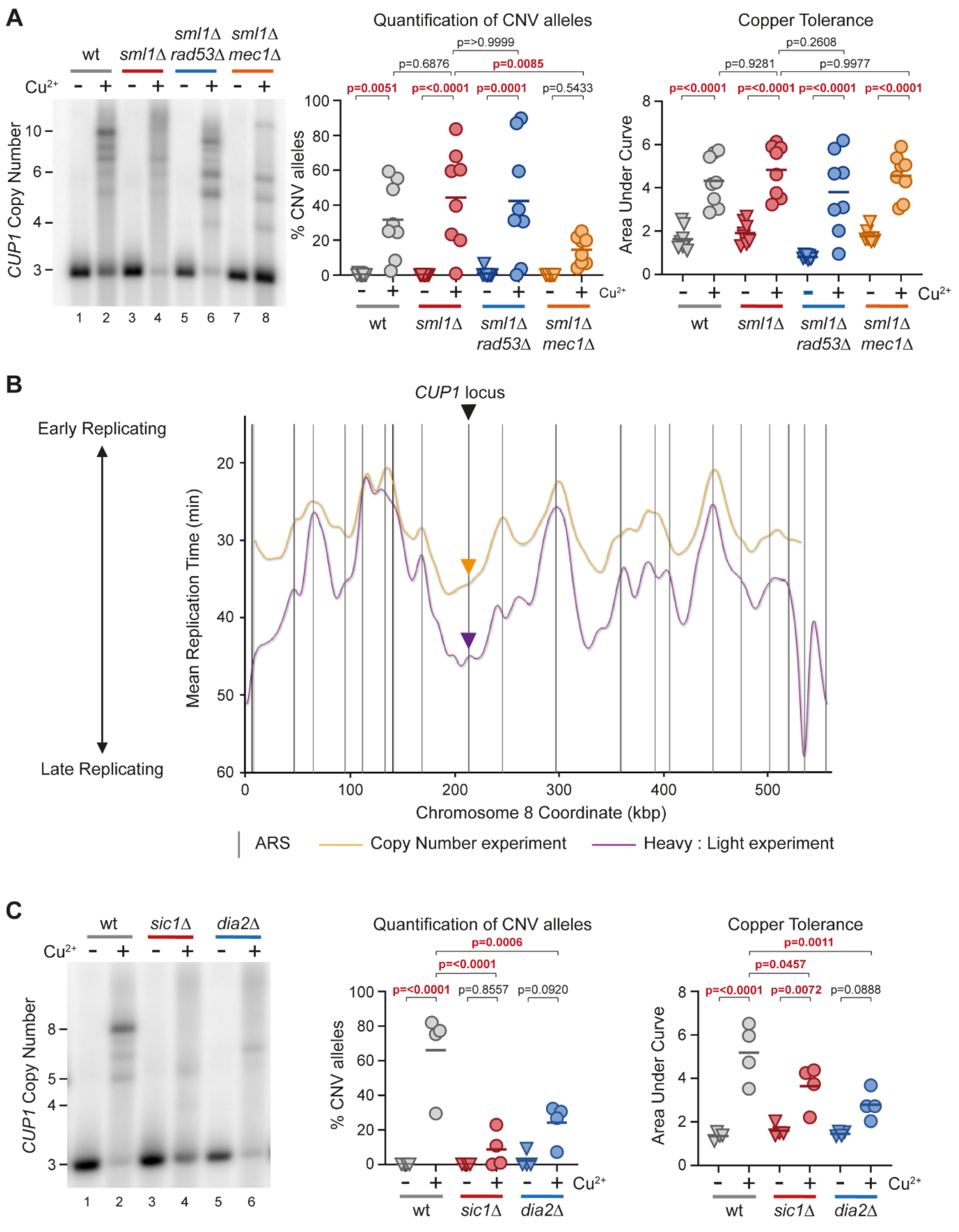

Supplementary Figure S5. Supplement to replication timing and fork progression control *CUP1* CNV. **(A)** Southern blot analysis of *CUP1* copy number and copper adaptation analysis of *3xCUP1* wild-type (wt), *sml1*Δ, *rad53*Δ *sml1*Δ and *mec1*Δ *sml1*Δ cells, performed as in Figure 6A, n = 8. **(B)** Replication timing on Chromosome 8 from OriDB (1), with data collected by two independent studies measuring change in copy number (2) (shown in yellow) or Heavy : Light isotope transfer (3) (shown in purple). Grey lines correspond to Replication origins and arrows indicate the late replication timing of the *CUP1* locus from the respective studies. **(C)** Southern and growth curve analysis of *3xCUP1* wildtype (wt), and indicated mutant grown for 10 generations ± 0.3 mM $CuSO_4$ as in Figure 6A; n = 4.

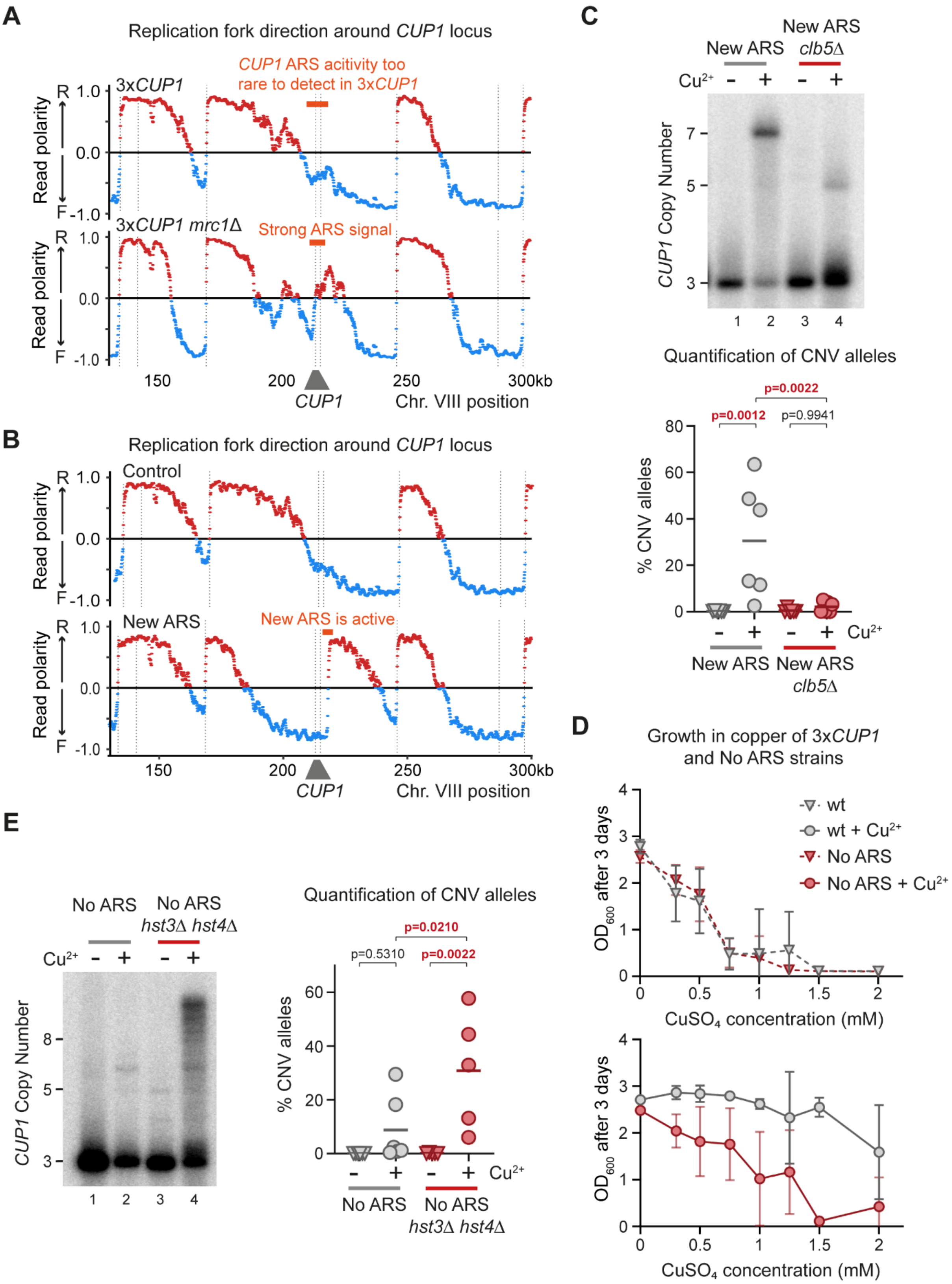
A
Replication fork direction around CUP1 locus
3xCUP1
CUP1 ARS acitivity too rare to detect in 3xCUP1
3xCUP1 mrc1Δ
Strong ARS signal
Read polarity
R 1.0
0.0
F -1.0
150
200
250
300kb
CUP1
Chr. VIII position
B
Replication fork direction around CUP1 locus
Control
New ARS
New ARS is active
C
New ARS
New ARS clb5Δ
Cu2+ - + - +
CUP1 Copy Number
7
5
3
1 2 3 4
Quantification of CNV alleles
p=0.0022
p=0.0012
p=0.9941
% CNV alleles
80
60
40
20
0
D
Growth in copper of 3xCUP1 and No ARS strains
wt
wt + Cu2+
No ARS
No ARS + Cu2+
OD600 after 3 days
CuSO4 concentration (mM)
E
No ARS
No ARS hst3Δ hst4Δ
8
p=0.0210
p=0.5310
p=0.0022

Supplementary Figure S6. Supplement to local replication origin firing regulates CNV at *CUP1* locus. **(A)** TrAEL-seq read polarity plots for 3x*CUP1* wild type and 3x*CUP1 mrc1*Δ strains, showing region surrounding *CUP1* on chromosome VIII, processed as Figure 7A, n=2. **(B)** TrAEL-seq read polarity plots for 3x*CUP1* control or New ARS strains (defined in Figure 7B), showing region surrounding *CUP1* on chromosome VIII, processed as Figure 7A, n=1. **(C)** Southern blot analysis of *CUP1* copy number for 3x*CUP1* New ARS (defined in Figure 7B), and 3x*CUP1* New ARS *clb5*Δ cells after 10 generations ± 0.3 mM $CuSO_4$, n=6. **(D)** Plots of final OD_{660} versus [$CuSO_4$] for wild type and No ARS cells that are naïve (above) or pre-cultured in 0.3mM $CuSO_4$ (below). Copper resistance of No ARS cells that have not been pre-cultured in $CuSO_4$ is similar to wild type, showing that deletion of this large region of the *CUP1* repeat does not impair the normal response to environmental copper. This is the source data underlying the plots in Figure 7D. **(E)** Southern blot analysis of *CUP1* copy number for 3x*CUP1* No ARS (defined in Figure 7B) cells, n=6, and 3x*CUP1* No ARS *hst3*Δ *hst4*Δ cells, n=5, after 10 generations ± 0.3 mM $CuSO_4$.

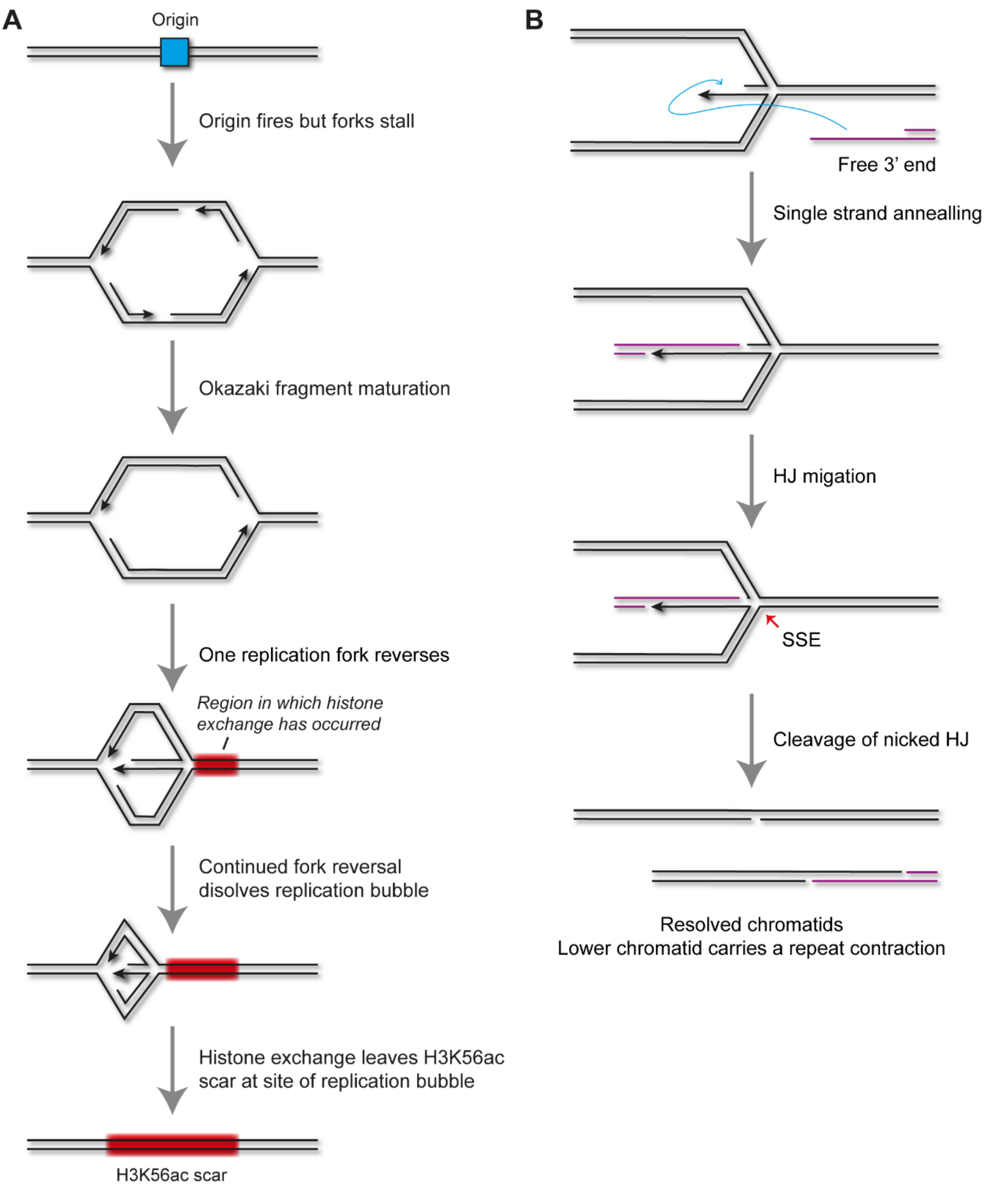

Supplementary Figure S7. Additional mechanisms. **(A)** Proposed mechanism for H3K56ac chromatin incorporation during unsuccessful replication origin firing. DNA strands are shown in black, the replication origin is shown in blue and chromatin containing H3K56ac shown in red. In the presence of topological stress, replication origins may fire but stall soon after. Positive supercoiling may promote reversal of such stalled fork structures to the point of dissolution, leaving behind a 'scar' of H3K56ac chromatin from initial chromatin assembly. **(B)** Resolution of single-ended DSB by reversed converging fork through single strand annealing. Stalling and reversal (possible with limited resection) of the converging fork provides an annealing target for the resected DSB. Migration provides a nicked Holliday Junction, the preferred substrate for Mus81, and resolution yields two complete chromatids, the lower of which as represented (stemming from the one ended DSB) can contain a repeat contraction.

Supplementary Tables

Tables are provided as Excel files.

S1 Table *S. cerevisiae* yeast strains used in this study

S2 Table Oligonucleotide pairs used in this study.

S3 Table Quantification of CNV derived from Mutant Screen. % CNV alleles were calculated as intensity of bands by southern blot analysis: CNV alleles / (CNV alleles + Parental allele) x 100. Fold-change in CNV was calculated as % CNV alleles for *gene*Δ / % CNV alleles of Wild Type.

Supplementary References

1. Siow, C.C., Nieduszynska, S.R., Muller, C.A. and Nieduszynski, C.A. (2012) OriDB, the DNA replication origin database updated and extended. *Nucleic Acids Res*, **40**, D682-686.
2. Yabuki, N., Terashima, H. and Kitada, K. (2002) Mapping of early firing origins on a replication profile of budding yeast. *Genes Cells*, **7**, 781-789.
3. Raghuraman, M.K., Winzeler, E.A., Collingwood, D., Hunt, S., Wodicka, L., Conway, A., Lockhart, D.J., Davis, R.W., Brewer, B.J. and Fangman, W.L. (2001) Replication dynamics of the yeast genome. *Science*, **294**, 115-121.
4. Brachmann, C.B., Davies, A., Cost, G.J., Caputo, E., Li, J., Hieter, P. and Boeke, J.D. (1998) Designer deletion strains derived from Saccharomyces cerevisiae S288C: a useful set of strains and plasmids for PCR-mediated gene disruption and other applications. *Yeast*, **14**, 115-132.
5. Dai, J., Hyland, E.M., Yuan, D.S., Huang, H., Bader, J.S. and Boeke, J.D. (2008) Probing nucleosome function: a highly versatile library of synthetic histone H3 and H4 mutants. *Cell*, **134**, 1066-1078.
6. Breslow, D.K., Cameron, D.M., Collins, S.R., Schuldiner, M., Stewart-Ornstein, J., Newman, H.W., Braun, S., Madhani, H.D., Krogan, N.J. and Weissman, J.S. (2008) A comprehensive strategy enabling high-resolution functional analysis of the yeast genome. *Nat Methods*, **5**, 711-718.
7. Hull, R.M., Cruz, C., Jack, C.V. and Houseley, J. (2017) Environmental change drives accelerated adaptation through stimulated copy number variation. *PLoS Biol*, **15**, e2001333.
